# Understanding equity-oriented maternity care for women of refugee background in high-income countries: a qualitative systematic review

**DOI:** 10.1186/s12939-026-02848-5

**Published:** 2026-04-20

**Authors:** Aishah Jameel, Nicole Pope, Fran Hearn, Amita Tuteja, Elisha Riggs

**Affiliations:** 1 Department of General Practice and Primary Care, The University of Melbourne, Melbourne, VIC Australia; 2 Policy and Equity, Murdoch Children’s Research Institute, Parkville, VIC Australia; 3 Monash Centre for Health Research & Implementation, Monash University, Clayton, VIC Australia

**Keywords:** Refugee background, Maternity care, Equity-oriented healthcare, High-income country, Qualitative systematic review

## Abstract

**Background:**

Women of refugee background in high-income countries experience disproportionately poorer maternity care experiences and maternal health outcomes compared to non-refugee women. Their maternity care needs remain under-recognised and under-researched.

**Aim:**

This qualitative systematic review aimed to synthesise evidence on (1) what women of refugee background need from maternity care in high-income countries, and (2) the barriers and enablers experienced by health, social, and community-based maternity care professionals providing maternity care to this population.

**Methods:**

Following the JBI methodology for systematic reviews, five databases were searched for peer-reviewed qualitative studies published in English between 2013 and 2025. Eligible studies were screened, data were extracted and appraised critically for methodological quality. Findings were synthesised using meta-aggregation and the overall confidence in synthesised findings were assessed using the JBI ConQual approach. The EQUIP framework was applied to the findings to ensure health equity considerations were captured.

**Findings:**

Fifty-six studies met the inclusion criteria. Three synthesised findings were generated: (1) Structural and systemic factors shape the provision of and access to equity-oriented maternity care; (2) Key components to equity-oriented maternity care are: trauma-informed care, culturally responsive care, woman and family-centred care, and continuity of care and carer; and (3) women’s settlement experiences influence their transition to new healthcare systems.

**Conclusion:**

Addressing the persistent health inequities faced by women of refugee background in high-income countries requires systemic reform, sustainable workforce investments and specialised training for care providers. Further research on the co-design of equity-oriented care interventions is also needed to ensure maternity services are responsive to the needs of this population.

**Supplementary Information:**

The online version contains supplementary material available at 10.1186/s12939-026-02848-5.

## Introduction

### Global context

Over the last three decades, over 123 million people worldwide have been forcibly displaced, with more than 40 million recognised as refugees, a record high [1]. The United Nations High Commissioner for Refugees (UNHCR) defines refugees as ‘persons outside their countries of origin who are in need of international protection because of feared persecution, or a serious threat to their life, physical integrity or freedom in their country of origin as a result of persecution, armed conflict, violence or serious public disorder’ [2]. (p.81) People seeking asylum are defined as ‘any person who is seeking international protection and has applied for refugee status or a complementary international protection status and has not yet received a final decision on their claim’ [3]. 

### Definition of refugee background

In this review, the term ‘refugee background’ is used to describe the study population and includes people who have been legally recognised as refugees, have a refugee-like background or are seeking asylum. There is no authoritative definition of the term ‘refugee background’ and this term was used in preference to the legal term ‘refugee’. This practice is adopted by a number of international and national refugee health and settlement organisations such as the Foundation House (Victorian Foundation for Survivors of Torture) [4] to acknowledge that some women may have migration histories that are shaped by conflict, persecution or humanitarian need, but may not meet the 1951 Refugee Convention definition or hold formal refugee status [28]. Refugee background included women who were: (1) formally recognised as refugees; (2) self-identified as having a refugee or refugee-like background; (3) arrived on a humanitarian visa; (4) seeking asylum; or (5) forcibly displaced from their home country due to war, violence, serious public disorder, or fear of persecution.

Although most people initially seek refuge in neighbouring low- and middle-income countries, permanent re-settlement occurs more frequently in high-income countries such as the United States of America, Canada, Australia, and Germany, together accounting for 94 per cent of all settlement arrivals in 2024 [1]. 

### Maternal and child health outcomes of refugee background communities

Women and girls represent half the population of refugees, and many are of reproductive age with ongoing maternity care needs. This has major implications for high-income countries with significant refugee settlement, where pregnant and mothering women of refugee background represent a particularly vulnerable group with complex health needs [5]. 

Compared to non-Indigenous women and children born in countries of settlement, women of refugee backgrounds and their babies experience disproportionately poorer maternal and child health outcomes, including higher rates of postnatal depression, preterm birth, miscarriages, stillbirth, and being small-for-gestational age [6–8]. Evidence further demonstrates that these early health disparities extend into later life, with children of refugee backgrounds experiencing higher risks of chronic disease in adulthood [9]. 

### Inequities in access, navigation, and uptake of health services

Antenatal attendance and under-utilisation of maternal health services are significant issues among women of refugee background. They are more likely to initiate antenatal care later in their pregnancy, or not at all, and are less likely to receive the recommended number of antenatal visits [10, 11]. These low levels of service use suggests the presences of multiple barriers to accessing maternity care [12, 13]. Although often grouped with other migrant populations, women of refugee background experience distinct and inequitable challenges compared to economic migrants and other socially and economically disadvantaged groups. These include coping with the psychosocial and health impacts associated with forced migration, war and persecution in countries of origin, language barriers, limited health literacy, racism and discrimination, and settlement challenges in a new country. Collectively, these factors hinder access to and engagement with maternity services [12, 14, 15]. Furthermore, many current models of maternity care in high-income Western countries such as Australia, UK and across Europe fail to adequately address the impact of forced displacement, psychological trauma, loss of support networks, and language and cultural needs of refugee women contributing further to limited service use and negative maternity care experiences [8, 13, 16–20]. 

### The role of health, social, and community-based maternity care providers

Health, social, and community-based maternity care providers are responsible for delivering safe, respectful, woman-centred, and high-quality maternity care to all women, including women of refugee background [21]. Although many providers strive to meet these needs, they often face significant barriers that can impact the care they are able to deliver [22]. 

In this review, healthcare professionals are defined as registered clinical providers that provide medical or clinical maternity care during pregnancy, labour and birth, and up to 12 months postpartum. These include midwives, obstetricians, general practitioners with shared care, and nurses. Social care professionals are defined as providers that provide social support during pregnancy, labour and birth, and up to 12 months postpartum. These include but are not limited to hospital social workers, mental health clinicians, refugee health nurses, and interpreters. Community-based maternity care professionals are the non-clinical maternity support workforce that work within the broader maternity care ecosystem and provide emotional support, advocate for women, and provide continuity of presence. These include doulas and bicultural workers.

Understanding the experiences of health, social, and community-based maternity care providers, particularly the barriers and enablers to providing maternity care, is essential for identifying how maternity services can better meet the needs of women of refugee background.

### Rationale for combining both perspectives

The experiences of women of refugee backgrounds receiving maternity care and the experiences of health, social, and community-based maternity care professionals providing that care are often examined in isolation within qualitative systematic reviews [12, 20, 22–30]. While this approach can allow for greater analytical depth within each group, it risks overlooking the relational and structural interplay between women’s maternity care needs and the realities of service provision experienced by health, social, and community-based maternity care professionals.

Synthesising both perspectives within a single review provides a more comprehensive and nuanced understanding of maternity care provision. In particular, this review highlights how women of refugee background’s maternity care needs intersect with the barriers and enablers experienced by health, social, and community-based maternity care professionals who provide care for them. Examining these perspectives together allows the identification of areas of alignment, tension, and missed opportunities, addressing an important and notable evidence gap. The focus on the *needs* of women, rather than their experiences alone, directly centres women as right-bearing individuals with legitimate entitlements to quality maternity care, and addresses the types of changes that need to occur within policy and practice.

Additionally, these intersecting perspectives are analysed through an equity lens, enhancing the conceptual strength of the study. Understanding the needs of women and experiences of health, social, and community-based maternity care providers through this lens offers insight into how structural, systemic, organisational, and individual-level factors within high-income countries shape the delivery of equitable and inequitable maternity care. This integrated, equity-oriented approach distinguishes this review from prior syntheses.

To our knowledge, this is the first comprehensive systematic review of qualitative evidence that synthesises both (1) the maternity care needs of women of refugee background living in high-income countries and (2) the barriers and enablers experienced by health, social, and community-based maternity care professionals providing care to this population, within a health and social equity lens.

### Review questions

This review systematically identified, explored, and synthesized qualitative studies on the maternity care needs of women of refugee background and provider perspectives by addressing the following questions:

1. What do women of refugee background need from maternity care in high-income countries?

2. What are the barriers and enablers for health, social and community-based maternity care professionals providing care to women of refugee background?

## Methods

This review followed the Joanna Briggs Institute (JBI) meta-aggregative approach to systematic reviews of qualitative evidence [31] and the protocol was registered on PROSPERO (CRD42023462543).

### Eligibility criteria

#### Type of studies

We included peer-review studies reporting original qualitative data and published in English. Eligible qualitative research methodologies included phenomenology, grounded theory, ethnography, and action research. Mixed methods studies (i.e. both qualitative and quantitative approaches) were included if the qualitative component were clearly identifiable and its methods and findings could be extracted independently. Only studies published between 2013 and 2025 were considered. We excluded narrative reviews, opinion pieces, commentaries, systematic and scoping reviews, meta-analyses, case reports, editorials, letters, study protocols, positions statements, conference proceedings, guidelines, and grey literature. Studies published in languages other than English were also excluded.

#### Population

The review focused on two groups: (1) women of refugee background and (2) health, social, and community-based maternity care professionals who provide care to women of refugee background during the perinatal period. 

We included studies involving women aged ≥ 18 years with lived experience of forced displacement, persecution, and/or human rights violations who had received maternity care.

Excluded were women born in the host country, Indigenous (including Aboriginal and Torres Strait Islanders, Native Americans, Inuit and Metis), migrants (including undocumented migrants), survivors of human trafficking, and other vulnerable women who did not meet the refugee-background definition. Survivors of trafficking and undocumented migrants were excluded as they represent distinct experiences with their own specific trauma profiles, legal statuses, and healthcare context, frequently lacking entitlements to publicly funded healthcare services in many high-income countries and experience unique risks associated with detection and deportation that are not comparable to the experiences of women of refugee backgrounds.

Studies including both migrant and refugee women perspectives were only included if the majority were of refugee background; if this could not be determined or results specific to the experiences of women of refugee background could not be isolated, the study was excluded.

We included studies involving health, social, and community-based maternity care professionals providing maternity services to women of refugee background. Community-based maternity care professionals, including doulas and bicultural workers, were included due to their recognised role within the maternity care ecosystem in supporting relational continuity and culturally responsive care.

Studies were excluded if participants worked exclusively with migrants, undocumented, Indigenous, or socioeconomically disadvantaged women without refugee-background experiences.

#### Phenomena of interest

We considered studies reporting the maternity care needs and experiences of women of refugee background, as well as experiences of professionals providing that care. Maternity care was defined as care delivered during pregnancy, birth, and up to 12 months postpartum.

#### Context

We considered studies conducted in maternity and postpartum care services provided in all settings in high-income countries (as classified by the World Bank) [32]. These included but were not limited to hospitals, clinics, and community health organisations.

### Search strategy

A comprehensive search strategy was developed in consultation with an experienced librarian. Five electronic databases were initially searched on 22nd August 2023: MEDLINE (Ovid), PubMed, CINAHL (EBSCOhost), Embase (Ovid) and Web of Science. Search terms were derived from relevant keywords and tailored to each database (Appendix A). The search was updated on 24th September 2025 to capture newly published studies. Reference lists of all included studies were manually screened for additional eligible publications.

#### Study Selection

**Fig. 1:**
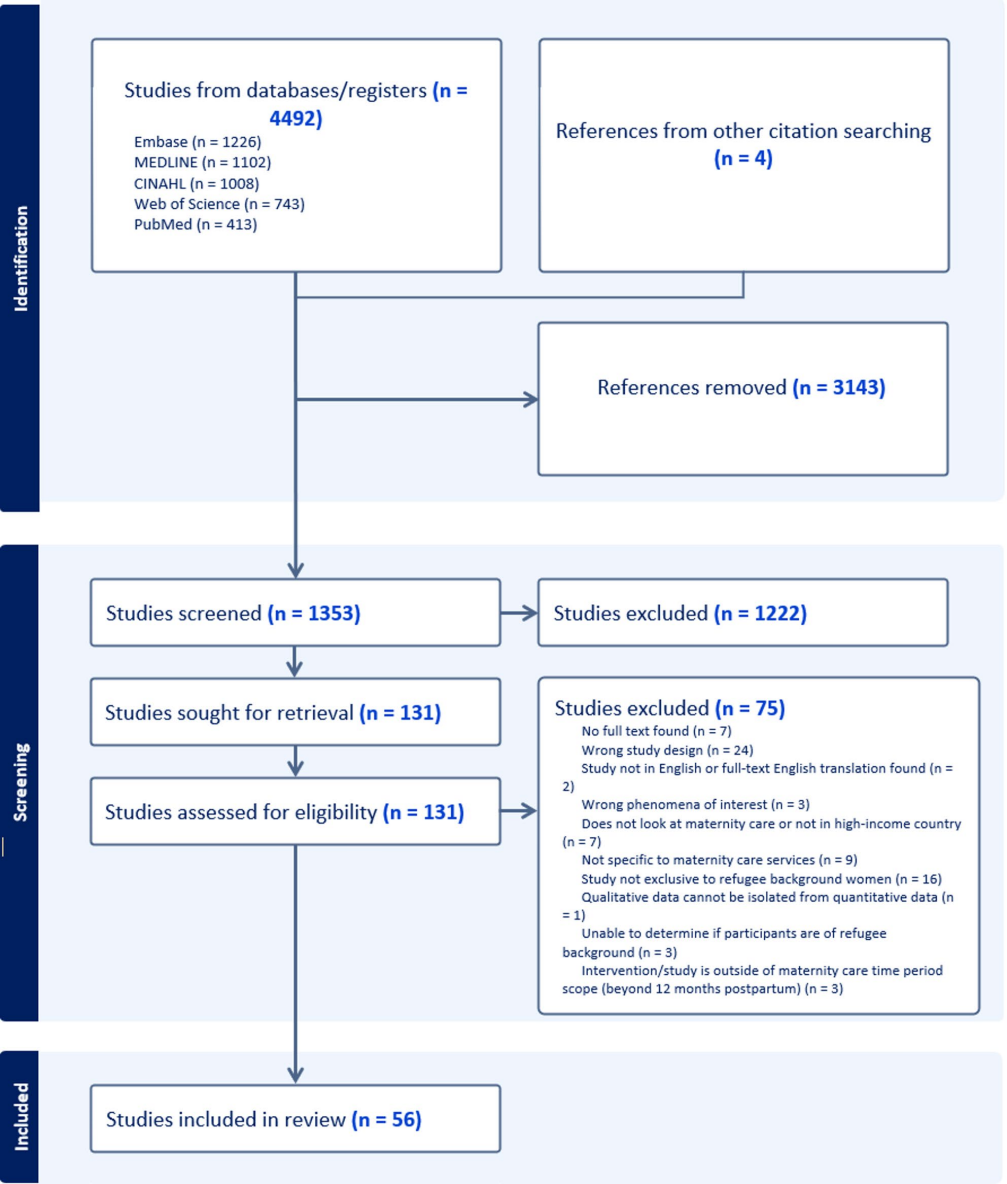
PRISMA flow diagram

All retrieved records were imported into Covidence [33] for de-duplication and screening. Screening against the inclusion criteria was conducted independently by two reviewers (AJ, FH, or AT). First by title and abstract where studies deemed potentially relevant were retrieved in full, followed by full text. Reasons for exclusion were recorded. Disagreements were resolved through discussion until consensus was reached. The overall selection processes were documented and presented in a Preferred Reporting Items for Systematic Reviews and Meta-analyses (PRISMA) flow diagram (Fig. 1).

### Assessment of methodological quality

The methodological quality of included studies were appraised using the JBI Critical Appraisal Checklist for Qualitative Research [34]. This tool evaluates congruity between study design, methodology, data collection, interpretation, reporting, ethical considerations, and representation of participants. Each item was graded as “Yes”, “No”, or “Unclear”. Although the checklist is commonly used to determine the credibility of studies and to inform inclusion decisions, in this review, all studies were retained regardless of their methodological quality. This decision was made to ensure that the breadth and richness of available studies could contribute to addressing the literature gap of maternity care needs of women of refugee background. See Results for a short narrative summary and Appendix B for more detail.

### Data extraction

The extracted data included citation details, aim, country, participant details, study design, methodology, methods, summary of findings, any reported health equity interventions, author interpreted findings, associated themes, descriptions, and illustrative participant quotes (Appendix C). To ensure accurate representation and trustworthiness, all findings were assessed for credibility and assigned a level: (1) Unequivocal (U) where findings were clearly supported by accompanying data; (2) Credible (C) where findings were supported but lacked detail or clear associations with data; and (3) Not Supported (NS) where findings were not substantiated by data reported. Data extraction was completed in Microsoft Excel by the primary reviewer (AJ), with 10% independently extracted by a tertiary reviewer (ER) to ensure accuracy and consistency.

### Data synthesis

Meta-aggregation was used to synthesise findings. The review team met in four videoconferences to list and colour-code all findings by study, group at least two findings of similar meanings into categories, develop categories names and descriptions, and iteratively review, re-group and refine categories until consensus was reached. Final categories were then aggregated into synthesised findings, each comprising at least two categories, which were drafted by the primary reviewer (AJ) and validated by the full review team. Women’s and providers’ perspectives were synthesised separately, then examined together to identify overlapping categories.

**Fig. 2:**
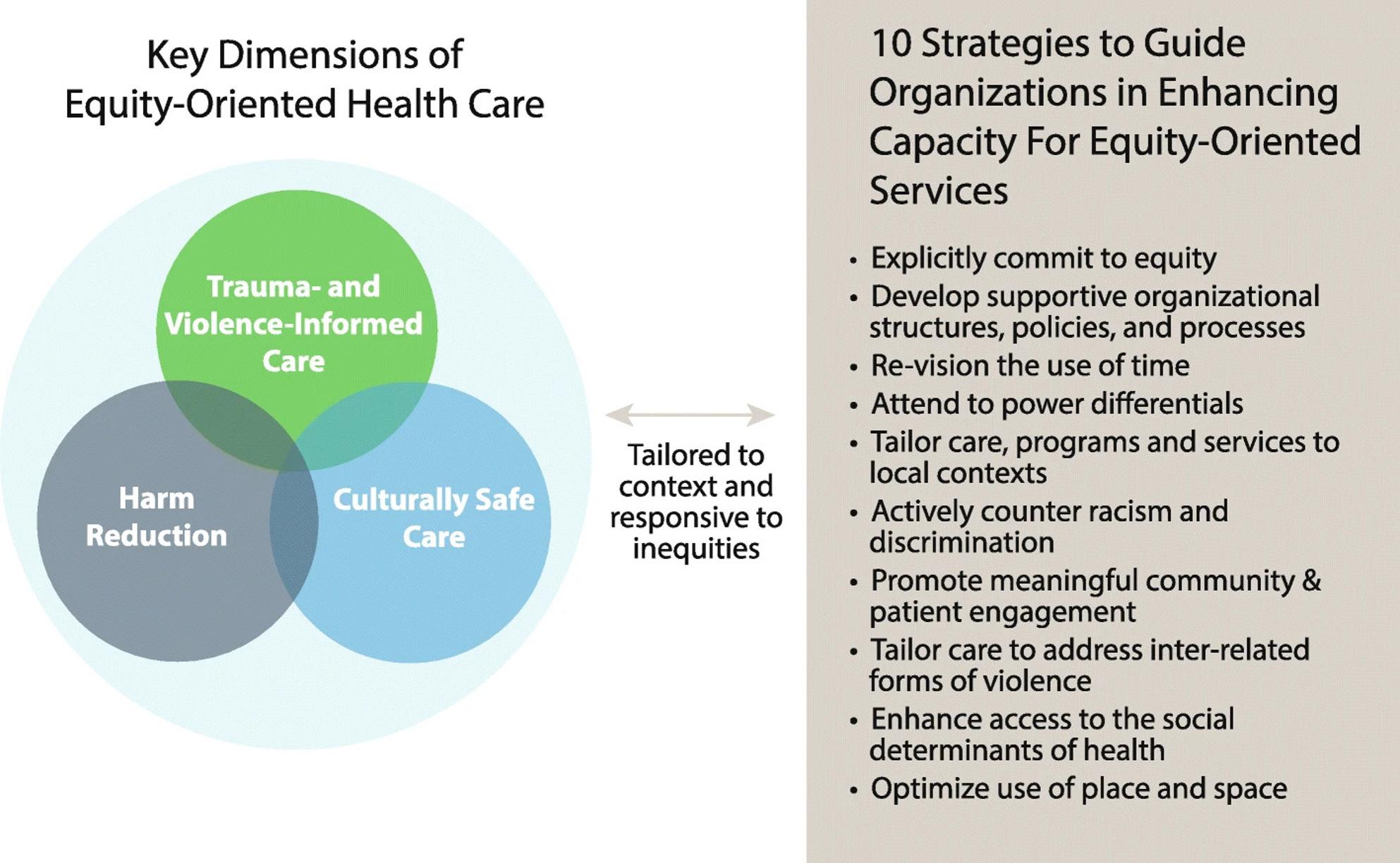
EQUIP framework reproduced from Browne et al. [35]

A health equity lens, guided by the EQUIP framework [35] (Fig. 2), was applied to the findings, providing a framework to examine the maternity care needs of women and the barriers and enablers experienced by health professionals. The EQUIP framework is a multi-component, evidence-informed health equity framework used to enhance the delivery of equity-oriented care to socially marginalised groups in healthcare settings [35]. In applying this framework, this review sought to assess whether the existing dimensions of EQUIP (i.e., trauma- and violence-informed care, harm reduction, and culturally safe care) sufficiently captured the unique maternity care needs of women of refugee background, or whether its dimensions required revision to more explicitly reflect these needs. Additionally, the barriers and enablers described by maternity care workforce were mapped against the 10 strategies to guide organisations to enhancing capacity for the provision of equity-oriented services. This allowed us to identify relevant strategies, where gaps remained, and areas for adaptations that reflect the challenges experienced by health and social care professionals caring for women of refugee background.

### Assessment of confidence in findings

The final synthesised findings was evaluated according to the JBI ConQual approach to assess the confidence in the findings produced from the review [36]. The ConQual approach is a system used to evaluate the confidence of synthesised findings through meta-aggregation developed by the JBI Qualitative Methodology Group [36]. The level of confidence (ConQual score) for synthesised findings is based on the dependability and credibility of the included studies and its associated findings. Each synthesised finding is initially assigned a ‘high confidence’ rating and is downgraded a level as necessary according to the dependability and credibility scores assigned.

## Results

### Characteristics of included studies

A total of 1,353 studies were identified in the search after the removal of duplicates; 56 studies met the inclusion criteria and were included in the review. See Fig. 1 for details on the search and screening process. The studies were published between 2013 and 2025, with most studies published in 2023 [13, 37–45]. No studies were published in 2019.

Eleven high-income countries are represented in this review, mainly from Australia [13, 17, 39, 40, 46–57], and the USA [37, 41, 42, 58–69]. Other countries include Canada [70–74], the UK [45, 75–78], Norway [38, 79–81], Poland [44, 82, 83], Ireland [84, 85], the Netherlands [43, 86], Finland [87], Germany [15], and Greece [88]. One multi-country study was conducted in Greece, Netherlands and the UK [89]. It is important to note that while all included studies were conducted in high-income countries, the organisation, funding, and delivery of maternity services varies considerably across these settings. For example, many countries in Europe, Australia, the UK, and Canada have universally funded public maternity care services, whereas the US relies on an insurance-based system.

Fifty-one studies were of qualitative design that adopted a range of approaches including participatory action research [37, 51, 54, 55, 57, 58, 68, 79], grounded theory [63, 70, 72], ethnography [37, 64, 71], phenomenology [15, 40, 48, 77], descriptive design [39, 42, 47, 53, 56, 58, 59, 65, 67, 69], and interpretative descriptive design [73]. Twenty-four qualitative studies did not report a specific research methodology [13, 17, 38, 41, 44–46, 49, 61, 62, 66, 74–76, 78, 80–88]. Four studies used a mixed -methods design where the qualitative component of the study could be isolated for the purpose of data extraction for this review [50, 52, 60, 89], and one study was a cross-sectional survey study that contained open-ended questions that were analysed using qualitative methods [43]. 

Most studies (*N* = 35) collected data through semi-structured interviews [15, 17, 37–40, 42, 44, 48, 50, 52, 56, 57, 59, 61, 62, 64–66, 69, 70, 72–74, 77–82, 84–87, 89], 11 studies undertook focus groups only [41, 47, 49, 53, 55, 60, 68, 71, 75, 83, 88], and seven utilised both methods [45, 46, 51, 54, 58, 63, 67]. One study collected data from a mixture of focus group discussions, semi-structured interviews and analysis of a narrative document [76], one undertook semi-structured interviews and a policy review [13], and one captured participant perspectives via an online survey containing open-ended questions [43]. 

Over half of the studies (*N* = 30) included the experiences of women from refugee and asylum seeker backgrounds [15, 37, 38, 41, 42, 45, 47–49, 51, 55, 57–60, 64–66, 68–72, 75, 77, 79–81, 84, 87]. Twenty reported on the perspectives of health and social care professionals [13, 39, 40, 43, 44, 46, 50, 53, 62, 63, 67, 73, 76, 78, 82, 83, 85, 86, 88, 89] and six studies reported on the perspectives of both women and service providers in the same paper [17, 52, 54, 56, 61, 74]. One study [54] included the perspectives of women, men and service providers, however, only the perspectives of women and service providers were extracted and deemed relevant for the review.

Participants were 523 women from diverse refugee and asylum seeker backgrounds, commonly from Somalia [17, 38, 42, 47, 52, 58, 60, 64, 65, 79, 80, 87], Afghanistan [41, 51, 52, 54, 56, 59, 66, 69, 87], Syria [15, 38, 41, 59, 66, 70, 72, 79], Myanmar [41, 48, 49, 55–57, 60, 61, 68] and Sudan [47, 48, 52, 56, 59, 71, 75]. Some of the women were new arrivals and had resided in countries of settlement for less than 9 weeks, while others had lived for up to 16 years. The women were between 18 and 55 years of age. Papers which have originated from the same study and collected data from the same group of participants have been accounted for to avoid duplicates [42, 65, 70, 72]. 

511 service providers were included in the review, comprising a range of health, social, and community-based maternity care professionals including midwives, midwifery students, doctors (general practitioners and obstetricians), nurses (refugee health, maternal and child health, women’s health, chronic disease, labour and delivery), nurse practitioners, doulas, managers (health service and midwifery), social workers, interpreters, mental health staff, and multicultural/bicultural health workers. The providers were aged between 20 and 70 years and had worked between six months and 38 years in the health service. They worked in urban and regional/rural settings, including maternity care facilities, refugee-specific health services, hospitals, community services, post-settlement agencies, and local health districts.

Fifteen studies implemented an equity-oriented intervention, which is described in this review as an intervention that helps to close the health and social equity gap while aiming to (1) improve care recipient experiences, (2) improve health and wellbeing outcomes, and (3) reduce healthcare costs [90]. These interventions could be a new model of care, policy or guideline, program or service, or new type of personnel implemented by the health service. The types of interventions identified included a Postpartum Peer Navigator Program [59], a refugee-specific Midwifery Group Practice service [46, 47], cultural competency training [89], Group Pregnancy Care [39, 49], community-based or multicultural doulas [38, 40, 63], Cross-Cultural Workers [50, 91], a community-based antenatal service [48], and specialist clinics for women of refugee backgrounds [52, 60, 73]. See Table 1 for more detail.

**Table 1:** Characteristics of included studies (*N* = 56)

Authors and Year of Publication	Country	Study aims/purpose	Study Design and Methodology	Equity-oriented intervention	Participants	Main findings
Agbemenu et al. (2021)	USA	To identify perceived protective mechanisms used to avoid obstetric interventions and the underpinning factors that influence aversion to obstetrical interventions by Somali refugee women	Qualitative: Descriptive, exploratory using CBPR. Interviews and focus groups	N/A	40 Somali women of refugee backgrounds	Four themes: Intentionally not seeking or misleading prenatal care; changing hospitals and/or providers when care has commenced; delayed hospital arrival during labour; outright refusal of care
Alsamman et al. (2025)	USA	To explore the postpartum experiences of refugee women and assess their interest in and opinions on a postpartum peer navigator program	Descriptive qualitative: Semi-structured interviews	Postpartum peer navigator program	26 women of refugee backgrounds from Syria, Afghanistan and Sudan	Barriers to postpartum health were identified such as lack of social support, stigmatization of mental health care, misinformation on postpartum contraception, and difficulties with interpreter use. Participants were interested in a postpartum navigator program.
Asbjornsen et al. (2025)	Norway	To explore the experiences of women with refugee backgrounds during pregnancy, birth and postpartum, focusing on their experiences of the maternal healthcare services	Qualitative: Feminist Participatory Action Research approach. Semi-structured interviews	N/A	10 women of refugee backgrounds from Eritrea, Syria, Lebanon, Kuwait, Liberia, Congo and Somalia	Having a refugee background, combined with diverse socioeconomic backgrounds and caregiving responsibilities, shaped women’s access to and engagement with maternal healthcare services in Norway. Structural barriers such as limited access to interpretation, limited social networks, and unfamiliarity with healthcare systems, resulted in emotional strain and inconsistent use of services.
Ayers et al. (2025)	USA	To explore community-based doulas’ experiences working with immigrant clients and maternal health-care providers	Descriptive qualitative: Semi-structured interviews and focus group discussions	N/A	8 doulas	Three themes: Experiences with immigrant clients; experiences with maternal healthcare providers; suggestions to improve support for immigrant clients
Banke-Thomas et al. (2017)	USA	To assess satisfaction of refugee women from multiple ethnic background in utilizing the specialised prenatal care provided at the Refugee Women’s Health Clinic	Mixed methods: focus group discussions	Refugee Women’s Health Clinic	Between 30 and 40 women of refugee backgrounds (exact number not specified). Languages spoken include Arabic, Kirundi, Swathi, Somali and Burmese	Four themes: Recognition of the importance of prenatal care; positive drivers for use of prenatal care; barriers to prenatal care; opinions of specialised prenatal care
Cameron et al. (2021)	Canada	To understand Syrian refugee women’s experiences accessing postnatal healthcare services and supports during the COVID-19 pandemic	Qualitative: Constructivist grounded theory. Semi-structured interviews	N/A	8 Syrian women of refugee backgrounds	Three themes: impacts of COVID-19 postnatal healthcare; loss of informal support; and anxiety and grief caused by COVID-19
Cameron et al. (2022)	Canada	To understand Syrian refugee women’s perceptions and experiences of access (barriers and facilitators) to formal health services and informal supports during the postpartum period and to identify valued and missing services and supports in the community that are needed	Qualitative: constructivist grounded theory. Semi-structured interviews	N/A	11 Syrian women of refugee backgrounds	Four themes: the importance of social support; impact of structural barriers on access to and quality of care; provider paternalism and women’s decision-making autonomy; and valued and missing services
Chrzan-Dętkoś & Murawska (2023)	Poland	To analyse midwives’ experiences of maternity care for females from Ukraine after the war and identify the main challenges of working with potentially vulnerable Ukrainians and the need for support for patients and midwives.	Exploratory qualitative: Semi-structured interviews	N/A	8 midwives	Five themes: the burden of traumatic stress on mothers and midwives; cultural differences; challenges in working with female patients from Ukraine; the need for additional support in their workplace; vital elements of working with people who have experienced war and those with whom we do not share a language
Coe et al. (2024)	Australia	To explore what culturally safe pregnancy care is to Karen women of refugee background	Qualitative CPBR study utilising photovoice: Focus group discussions.	N/A	5 Karen women of refugee backgrounds	Three themes: building foundations for belonging; cultivating reciprocal curiosity; storytelling as an expression of self and shared power
Denzongpa & Nichols (2023)	USA	To explore the maternal accounts of resettled Bhutanese refugee women who have maternal healthcare experiences in the US and Bhutan.	Qualitative: CBPR focused ethnographic study utilising transnational feminist approach through a decolonial lens. Semi-structured interviews	N/A	13 Bhutanese women of refugee backgrounds	Themes identified include women’s experiences of familial support, impact of setting-based maternal health access, and quality of care experienced.
Dube et al. (2024)	Australia	To explore and describe women’s experiences and perceptions of care from a dedicated Refugee Midwifery Group Practice service.	Qualitative exploratory descriptive: Focus group discussions	Refugee Midwifery Group Practice service	16 women of refugee backgrounds from Somalia, Sudan, Kenya, Congo, Sierra Leone, and Ethiopia	Four themes: accessibility of care; women feeling accepted; value of relationality; service expansion and promotion. Women had positive experiences and acceptability due to easy access, strong woman-midwife relationships, and culturally safe care.
Dube et al. (2025)	Australia	To explore and describe midwives’ experiences and perceptions of working at a dedicated Refugee Caseload Midwifery Group Practice service.	Qualitative: Focus group discussions and interviews	Refugee Midwifery Group Practice service	8 midwives	Three themes: accessible and responsive care; understanding and valuing women’s needs; strong partnerships.
Due et al. (2022)	Australia	To understand the relationship between psychological wellbeing and perinatal care amongst African refugee women and identify potential improvements to perinatal healthcare services to promote positive psychological outcomes	Qualitative: semi structured interviews	N/A	19 women of refugee backgrounds from various African countries and 20 service providers	Four themes: continuity of care and relationships; culturally and refugee responsive care; women as equal decision-makers in their perinatal care; and perinatal healthcare experiences have long-lasting wellbeing implications
Erga-Johansen & Bondas (2023)	Norway	To illuminate immigrant women’s experiences of multicultural doula care during pregnancy and childbirth	Qualitative: Semi-structured interviews	Multicultural doula	7 women of refugee backgrounds from Syria, Somalia and Ethiopia	Four themes: feeling alone and scared – safeguarded by the multicultural doula; needing to be looked after – cared for by the multicultural doula; not understanding the language – understanding with the multicultural doula; giving birth in a new and unfamiliar culture – the multicultural doula as a guide with the midwife
Evans et al. (2022)	UK	To ask what refugee women want from maternity care, and to understand how to tailor services, and to ensure they are acceptable to refugee women.	Qualitative: Focus group discussions	N/A	10 women of refugee and asylum seeker backgrounds from various countries	Three themes: feeling safe in the maternity system and in their communities; fair and equal access and treatment in maternity care and the asylum system; and building a future in the UK
Fair et al. (2021)	Greece, Netherlands, and UK	To evaluate the impact of the ORAMMA training and explore midwives’ experiences of the training and providing care to women within the ORAMMA project	Mixed methods: Questionnaires and semi-structured interviews	Cultural competence training	12 midwives	Three themes of training experience: appropriate and applicable; made a difference; and training gaps 3 themes of experience caring for women: supportive care; working alongside peer supporters; challenges faced
Gateri (2024)	Canada	To understand the experiences of women refugee claimants and their access to pre- and postnatal services	Qualitative: Semi-structured interviews	N/A	8 women of refugee backgrounds from various African countries and 6 service providers	Two themes: lack of health coverage; discrimination
Glavin & Sæteren (2016)	Norway	To explore Somali new mothers’ experiences with the Norwegian health care system and their experienced needs during the hospital stay and the postpartum period	Qualitative: semi-structured interviews	N/A	10 Somali women of refugee background	Four themes: inadequate integration into Norwegian society; need for and fear of a caesarean delivery; family support around the postpartum period; support from health services
Haith-Cooper & Bradshaw (2013)	UK	To explore midwifery students’ perceptions of how to approach the care of pregnant women seeking asylum	Qualitative: focus group discussions and semi-structured interviews	N/A	11 midwifery students	Despite learning to adopt a holistic, woman centred approach to midwifery care in the educational setting, once in clinical practice, some students appear to be influenced by underpinning medical and managerial discourses when approaching care decisions around the pregnant woman seeking asylum.
Hearn et al. (2023)	Australia	To explore professional staff experiences of implementing and facilitating a multidisciplinary equity-oriented model of Group Pregnancy Care for women of refugee background.	Exploratory descriptive qualitative: constructivism. Semi-structured interviews	Group Pregnancy Care	23 service providers	Five themes: knowledge sharing; bicultural family mentors- the critical link; finding our own ways of working together; power dynamics at the intersection of community and clinical knowledge; and system capacity for change
Hearn et al. (2024)	Australia	To understand how women of refugee background accessed health information and maternity and/or early parenting care during the COVID-19 pandemic	Exploratory descriptive qualitative: Constructivism. Semi-structured interviews	N/A	17 women of refugee backgrounds from Myanmar, Sudan, South Sudan, Iraq, Jordan and Afghanistan	Three themes: Structural inequities and the toll of the pandemic; supportive infrastructure; cultural safety during the pandemic
Henry et al. (2020)	Germany	To investigate how premigration experiences, conceptions about pregnancy and childbirth, health literacy and language skills influence access to health care, experiences of health care, and childbirth	Qualitative: Phenomenological. Semi-structured interviews	N/A	12 women of refugee backgrounds from Iraq, Syrian and Palestine	Conceptions of pregnancy and childbirth and premigration experiences influence women’s behaviours and experiences of pregnancy and childbirth. They contribute to barriers in accessing health care and lead to negative health outcomes.
Higginbottom et al. (2013)	Canada	To map out the experiences of immigrant Sudanese women receiving maternity care services throughout the prenatal period, and to explore the disparities, based on their ethnocultural beliefs.	Qualitative: Focused ethnography. Focus group interviews	N/A	12 Sudanese women of refugee backgrounds	The women mostly avoid anything that they believe could harm themselves or their babies. Pregnancy and delivery were strongly believed to be natural events without need for special attention or intervention. The sub-Saharan culture supports the dominance of the family by males and the ideology of patriarchy. Pregnancy and birth are events reflecting a certain empowerment for women, and the women tend to exert control in ways that may or may not be respected by their husbands. Individual choices are often made to foster self and outward perceptions of managing one’s affairs with strength
Khaw et al. (2023)	Australia	To explore the roles of community-based doulas in providing culturally responsive care to migrant women in maternity services, from the perspectives of maternity care providers and doulas	Qualitative interpretative phenomenological using a social constructivist epistemology and interpretative phenomenological approach: Semi-structured interviews	Community-based doulas	30 service providers	Doulas were seen to support and enhance migrant women’s maternity care experiences through numerous ways, strengthening cultural-responsive care provision. Doulas provided: continuous individualised support, social connectedness, created safe spaces, acted as cultural facilitators, provided non-judgemental support, enhanced communication and rapport with providers and made connections
Kirkdendall & Dutt (2023)	USA	To examine how contextual factors shape pregnancy and childbirth experiences for women	Qualitative using reproductive justice framework: focus groups	N/A	13 women of refugee backgrounds from Syria, Afghanistan, Myanmar and Congo	Three themes identified: isolation and alienated knowledge; gendered disparities and structural inequities; and community support and precarity
LaMancuso et al. (2016)	USA	To study the perspectives of Karen refugee women, their medical providers, and Karen interpreters/doulas on perinatal care for Karen women in resettlement.	Qualitative with social contextual model as the theoretical framework: semi-structured interviews	N/A	14 Karen women of refugee backgrounds and 14 Karen key informants and service providers	Karen women expressed gratitude for and understanding of perinatal care in Buffalo, and providers described Karen patients as agreeable but shy. Karen doulas offered an alternative view that exposed women’s many questions and concerns, and described how doula training empowered them as patients’ advocates. Low self-efficacy trauma histories, and cultural expectations may contribute to Karen women’s seeming agreeability. Doulas/interpreters possess insider knowledge of women’s concerns and facilitate communication between patients and the care team.
Lazar et al. (2013)	USA	To explore HCP perceptions of barriers to providing health care services to Somali refugee women, specifically to obtain info about providers’ experiences, training, practices and attitudes surrounding prenatal care, delivery, and management of female genital cutting	Qualitative: semi-structured interviews	N/A	14 service providers (5 men and 9 women)	Four themes: challenges in patient-provider communication; frustration with perceived Somali women’s resistance to obstetric interventions; providers’ perception of mistrust by their patients; suboptimal provider training in the care and management of women with female genital cutting.
Leoniuk et al. (2025)	Poland	To analyse the experiences of midwives providing perinatal care to war refugees	Qualitative: semi-structured interviews	N/A	16 midwives	Four groups of barriers in care were identified: Language, cultural, educational, and psychological
Lephard & Haith-Cooper (2016)	UK	To explore the maternity care experiences of local pregnant asylum-seeking women	Qualitative interpretative approach: Phenomenological. Semi-structured interviews	N/A	6 women of asylum seeker backgrounds from sub-saharan Africa and Eastern Europe	Five themes: pre-booking challenges; inappropriate accommodation; being pregnant and dispersed; being alone and pregnant; and not being asked or listened to
Lillrank (2015)	Finland	To explore how refugee women experience pregnancy and childbirth in interaction with Finnish maternity care professionals.	Qualitative: Semi-structured interviews (with interpreters present). Fieldnotes also taken.	N/A	11 women of refugee backgrounds from Somalia, Russia, Iran and Afghanistan	Three themes: good experiences and emphasizing satisfaction; dramatic experiences and disappointments with maternity care; and tragic experiences as a result of failures of the maternity care system
Lukasse et al. (2025)	Norway	To explore how newly arrived refugee women from Ukraine experienced maternity care in their new host country, Norway	Qualitative: semi-structured interviews	N/A	8 Ukrainian women of refugee backgrounds	Three themes: healthcare in country of origin; high quality care in new country; and challenges of being a refugee
Mendel et al. (2021)	USA	To explore pregnant refugee women’s experiences and perceived needs while navigating the US maternity care system from the perspectives of doulas	Qualitative using grounded theory: focus groups and semi-structured interviews	Doulas	13 doulas	Three themes: cross-cultural comparisons that contextualize how pregnant refugee women engage with the US maternity-care system; challenges women face in navigating the system and other systems; and the role of community supports in facilitating navigation
Missal et al. (2016)	USA	To explore Somali immigrant new mothers’ experience of childbirth in Minnesota	Qualitative: ethnonursing. Semi-structured interviews	N/A	12 Somali women of refugee background	Six themes: limitations of support due to separation from family; the importance of cultural and religious practices; the desired relationships with nurses; fear of caesarean section; value of education for Somali women; views of postpartum blues/depression
Nenko et al. (2024)	Poland	To explore the experiences and strategies of midwives providing maternity care to Ukrainian refugee women in Poland	Qualitative: Focus group interviews	N/A	32 midwives	Two themes: Challenges with caring for migrant women; Making it work: midwives strategies in facilitating better care
Njenga (2022)	USA	To explore Somali refugee women’s experiences and perceptions of Western maternity care in the US	Qualitative descriptive design: semi-structured interviews	N/A	15 Somali women of refugee background	Six themes: communication and resource provision; participatory decision-making; provider attitudes to cultural practices; understanding the US health care system; mistrust of Western health care; and religious beliefs
Njenga (2023)	USA	To explore the cultural beliefs and practices of resettled Somali refugee women around pregnancy and childbirth	Qualitative descriptive design: Questionnaire and semi-structured interviews	N/A	15 Somali women of refugee background	Cultural beliefs and practices influence women’s conceptualizations and conduct of pregnancy and childbirth. 4 subthemes: pregnancy is not illness; female traditions; keeping it secret; and keeping the faith
Olcoń et al. (2023)	Australia	To capture the experiences of maternal health service providers working with refugee and migrant women and their perceptions of the challenges faced	Qualitative: semi-structured interviews and policy review	N/A	16 service providers	Four themes: understanding the needs, experiences and identities of refugee and migrant women; improving access and quality of services; taking an individualised approach; and using interpreters
Owens et al. (2016)	Australia	To understand the lived experiences of migrant women of CALD backgrounds who had used a community-based antenatal service specialising in maternity care for multicultural women and explore whether using this service enhanced their perception of pregnancy care	Qualitative: Social constructionism epistemology using phenomenological theoretical framework. Semi-structured interviews	Community-based antenatal service	12 women, with 7 women of refugee backgrounds from Sudan and Myanmar	Four themes: social support; gaining knowledge; a holistic service; and new opportunities
Papadakaki et al. (2021)	Greece	To identify the barriers faced by service providers in the provision of quality care to refugee and migrant women	Qualitative: Focus group discussions	N/A	25 service providers	Four themes identified: low capacity to meet the health care needs of migrants in a culturally appropriate manner; doctor-centred system with minimal investment in the healthcare team; lack of service integration and continuity of care; low engagement during crisis – service providers’ burn out
Pierce et al. (2025)	UK	To understand the experiences of healthcare professionals delivering care to refugees, asylum seekers and undocumented migrants	Qualitative: Semi-structured interviews	N/A	22 Service providers	Two themes: pressures on service providers in delivering care; how service providers adapt care to navigate challenges
Riggs et al. (2017)	Australia	To explore the experiences of resettled Karen women attending group pregnancy care	Qualitative: Focus groups	Group Pregnancy Care	19 Karen women of refuge background	Four themes: learning together – informed, prepared, confident, and reassured; social and emotional support – sharing stories and experiences; trusting relationships – continuity of care and care provider; and challenges in the hospital – communication and privacy
Rogers et al. (2021)	Australia	To explore the perceptions of service providers regarding the CCW service and identify recommendations for improvement.	A sequential explanatory mixed methods study consisting of surveys and semi-structured interviews	Cross Cultural Worker service	19 service providers	Cross cultural Workers improved access to heath and community-based services and healthcare experiences for women and families by acting as a “bridge to heath” through the provision of culturally responsive care. However, there were organisational factors that affected the CCW service provision
Rowe et al. (2023)	UK	To explore the impact of the asylum-seeking process, understanding of wellbeing, expressed health needs, and the experiences of maternity care of women seeking asylum during pregnancy and after childbirth.	Qualitative: Focus group discussions and semi-structured interviews	N/A	14 women of asylum-seeking backgrounds from Nigeria, Albania, Egypt, Pakistan, Iran, Georgia, and Namibia	Four themes: women’s understanding of health and wellbeing during pregnancy and after childbirth; women’s specific health needs: cultural and language challenges; the experiences of women seeking maternity care in terms of maternal multimorbidity; support from health and social care provider systems and charitable organisations
Russo et al. (2015)	Australia	To explore the experiences of Afghan women living in Melbourne throughout pregnancy, birth, and early motherhood, and gain insight into the aspects of their experiences that they perceive as positively and negatively impacting their emotional wellbeing	Qualitative: feminist and sociocultural paradigms, and participatory community development values and principles. Focus groups and interviews	N/A	38 Afghan women of refugee background	Seven themes: satisfaction with services; interaction with health staff; barriers to seeking support in formal settings; family and female kin; culture, traditions, and community; changing roles of men; and enhancing connection to improve emotional wellbeing.
Smith et al. (2025)	USA	To explore the birthing experiences of South Asian refugees and identify what constitutes RMC for this unique population.	CBPR qualitative: focus group discussions	N/A	15 women of refugee backgrounds from Myanmar and Nepal	Five themes: interpersonal caring; flaws in US maternity care are amplified for refugees; multidimensionality affects knowledge, preferences, and expectations; complexity of the US health system combined with unfamiliarity contributes to lack of confidence; problems with language interpretation
Stapleton et al. (2013)	Australia	To explore the maternity care experiences of women who attended a specialist antenatal clinic	Mixed methods evaluation: semi-structured interviews conducted for qualitative study component	Specialist antenatal clinic	18 women of refugee backgrounds from Somalia, Sudan, Afghanistan, Burundi and Liberia Number of service providers interviewed not reported	The clinic was highly regarded by all participants. Continuity of care throughout the antenatal period was particularly valued by newly arrived. Positive experiences decreased as women transitioned from the clinic to labour and postnatal wards where they reported that their traditional birthing and recuperative practices were often interrupted by the imposition of Western biomedical notions of appropriate care. The centrally located clinic was problematic, frequently requiring complex travel arrangements. Appointment schedules often impacted negatively on traditional spousal and family obligations.
Tankink et al. (2024)	Netherlands	To gain insights from the experiences of Dutch midwives to inform and enhance the provision of tailored and equitable care for forcibly displaced women	Qualitative: semi-structured interviews	N/A	11 midwives	Barriers at midwife-women interactions included: language and interpreters; cultural differences; building trust; relocations of asylum seekers; delays in access to care; interdisciplinary collaboration; housing conditions; resettlement after forced migration; prevalent mental health issues. There is an imbalance between midwives’ responsibilities to care for women and the resources available
Tobin & Murphy-Lawless (2014)	Ireland	To explore midwives’ perceptions and experiences of providing care to women in the asylum process and to gain insight into how midwives can be equipped and supported to provide more effective care to this group in the future	Qualitative: interviews	N/A	10 midwives	Five themes: barriers to communication; understanding cultural differences; challenges of caring for women who were unbooked; the emotional cost of caring; and structural barriers to effective care
Tobin et al. (2014)	Ireland	To gain insight into women’s experiences of childbirth in Ireland while in the process of seeking asylum.	Qualitative: Unstructured interviews	N/A	22 women of asylum seeker backgrounds from Nigeria, Cameroon, Burundi, South Africa, Zimbabwe, Iran, Iraq, Zaire and Sierra Leone	The lack of shared language, communication, connection and culturally competent care had a subsequent impact on provision of adequate care. There was an apparent lack of education and preparedness of staff. The barriers (language and communication) appear to have been complicated by the medicalised birthing environment which heightened the women’s sense of isolation, fear and vulnerability.
Toke et al. (2024)	Australia	To explore Karen women’s perspectives of trauma- and violence informed pregnancy care.	CBPR qualitative: Semi-structured interviews	N/A	7 Karen women of refugee backgrounds	Three themes: Care design and accessibility; promoting choice and control; trauma-informed interpreting
Verschuuren et al. (2023)	Netherlands	To identify challenges and target areas for improvement of community midwifery care for asylum seekers and refugees with a residence permit	Cross sectional study with open-ended questions	N/A	102 community care midwives	Five themes: interdisciplinary collaboration; communication with clients; continuity of care; psychosocial care; and vulnerable situation
Willey et al. (2018)	Australia	To explore service provision for Victorian regional refugee families from the perspective of maternal and child health nurses and identify whether there are continuing professional development needs and MCH nurses who work with families from a refugee background	Descriptive qualitative: focus groups	N/A	26 maternal and child health nurses	Four themes: how to identify women from a refugee background; the MCH nurse role when working with families from a refugee background; interpreting issues; and access to other referral agencies
Winn et al. (2018)	Canada	To understand the experiences of healthcare professionals caring for pregnant refugee women	Qualitative: semi-structured interviews	Specialised refugee clinic	10 service providers	Five themes: pregnant refugees are a heterogeneous population facing multiple barriers to care; healthcare professionals specialized in refugee health engage in diverse strategies of care; funding cuts created a confusing system which jeopardized care; Syrian influx created additional strains on existing problems; and heath care professionals unfamiliar with refugee health may be overwhelmed
Worabo et al. (2024)	USA	To understand Afghan women’s maternal health experiences as a step toward designing culturally sensitive care.	Descriptive qualitative: semi-structured interviews	N/A	20 Afghan women of refugee background	Three categories: maternal healthcare experiences: pregnancy, birthing and postpartum; Communication: language barrier, relationship with husband and health information seeking; accessing to care: transportation and financing healthcare
Yelland et al. (2014)	Australia	To explore the responsiveness of health services to the social and mental health of Afghan women and men at the time of having a baby.	CBPR qualitative: interviews and focus groups	N/A	16 Afghan women of refugee background and 34 service providers	Afghan women and men reported significant social hardship during the period before and after having a baby in Australia but were rarely asked about their social health by maternity and early childhood services. Most health professionals recognised that knowledge and understanding of their client’s migration history and social circumstances was relevant to the provision of high-quality care. However, inquiring about refugee background, and responding to non-clinical needs of refugee families was challenging for many health professionals.
Yeo et al. (2024)	USA	To explore the factors influencing maternal healthcare access and utilization among Muslim refugee women resettled in the US.	Qualitative: semi-structured interviews	N/A	17 women of refugee backgrounds from Iraq, Syria and Afghanistan	Six themes: perceptions toward hospital and prenatal care; life skills; language proficiency; cultural norms and practices; social support and network; the characteristics of heath care providers

#### Results of methodological quality assessment

In all studies, there was congruity between the research methodology, research question, methods used to collect data, representation and analysis of data, interpretation of results, and evidence of ethical approval. Less than half of the papers addressed the influence of the researcher on the research and included a statement that located the researcher culturally or theoretically, and most studies did not report a philosophical perspective adopted in the study (Appendix B).

### Review findings

Three synthesised findings were produced from 268 findings and 12 categories (Appendix D):

1) Structural and systemic factors shape the provision of and access to equity-oriented maternity care.

2) Key components to equity-oriented maternity care: trauma-informed care, culturally responsive care, woman and family-centred care, and continuity of care and carer.

3) Women’s settlement experiences influence their transition to new healthcare systems.

Using the ConQual assessment tool, over 85% of the findings were assessed as “unequivocal” (U), 11.9% were graded as “credible” and 2.6% were graded as “not supported” (NS). The confidence levels for all three synthesised findings were rated as moderate, with dependability rated as high and credibility rated as moderate (Appendix E).

#### Structural and systemic factors shape the provision of and access to equity-oriented maternity care

This synthesised finding was generated through the aggregation of four categories, comprising 122 findings. Healthcare professionals and women of refugee background face a range of structural and systemic enablers and barriers that influence the provision and access of culturally responsive and equitable maternity care. These factors are interrelated and span individual, interpersonal, organisational, and policy levels, shaping care experiences in complex ways. An example of an enabler reported by women was access to specialised refugee health clinics. These services were highly valued by women as they provided continuity of care and carer and embodied a holistic and flexible approach to care delivery.

However, women of refugee background experienced complex, intersectional challenges after settlement that created multiple barriers to accessing equitable maternity care. Some of these challenges are the result of broader structural and systemic barriers such as national housing and migration policies and health care coverage. One of the main barriers identified were strained, over-capacity and inadequately funded health systems which impacted the abilities of health professionals to provide equitable care to women of refugee background. Health professionals reported having limited time, resources, and capacity to deliver adequate care and health information, contributing to the mismatches between service demand and delivery. This ultimately took a toll on their emotional and mental load.

*When caring for forcibly displaced women, you*

*feel like a social worker, you feel like a planner, you*

*feel like someone’s buddy*,* you feel like a psychologist* .

*– you just have a lot more roles than only your profession.*

*(Midwife 3* [86]*).*

Additionally, healthcare professionals who worked in mainstream facilities often lacked cultural awareness, knowledge, and appropriate skills to address the unique and complex health and psychosocial needs and cultural customs of women of refugee backgrounds, due to insufficient specialised training. This led to them feeling underprepared and unconfident to care for the women and offer appropriate support.

The main structural and systemic barrier shared between healthcare professionals and women was rooted in language and communication. Most health professionals did not speak the same language as the women, leading to communication challenges and the need for interpreter-mediated appointments. However, both groups consistently reported the lack of access to professional interpreters within services, with women also expressing the need for antenatal and postpartum care resources in their native languages. Where interpreters were provided, challenges were reported such as misinterpretation, not relaying all the information conveyed (potentially due to challenges translating complex medical concepts where equivalent terms in the women’s native language did not exist) and the presence of male interpreters which made women uncomfortable discussing their medical issues.

*I experienced difficulties with the interpreter*

*when I went to the obstetrician. When I asked for*

*an interpreter, they were all male. I felt shy and not*

*know how to talk to him. I was also not able to tell*

*the interpreter that I wanted a female interpreter. I* .

*found this very difficult.*

*(Woman from Syria*,* Age 35* [59]*).*

*Sometimes because when we talk with your OB (obstetric) doctor about some examination*,* so it’s difficult to tell them (male interpreters) anything free. So sometimes I feel reluctant to share some of the things*. *(Woman from Afghanistan* [66]*)*,

As illustrated by the above quotes, women have a strong preference for being cared for by female health, social, and community-based care professionals, including interpreters and doctors. When women are unable to express their preferences or advocate for their needs, this fosters negative experiences of care and has the potential to affect women’s future access to maternity services, resulting in long-lasting implications for the health and wellbeing of themselves and their child. Some health professionals were able to overcome some of the communication hurdles by employing diverse and innovative communication strategies such as using non-verbal visual aids and tools when professional interpreters were unavailable.

#### *Key components to equity-oriented maternity care are: trauma-informed care*,* culturally responsive care*,* woman and family-centred care*,* and continuity of care and carer*

This synthesised finding was generated through the aggregation of five categories, comprising 114 findings. Approaches to maternity care systems that incorporate models of continuity of care and carer, trauma-informed care, culturally responsive care, and woman and family-centred care, strengthen communication and foster trusting, supportive relationships between women and their healthcare providers. In addition to the desire for timely and accessible care, women valued healthcare providers that took the time to listen to their stories, concerns and needs, and involved them in the decision-making process concerning their health and the health of their baby. Women reported feeling heard and safe, had a sense of choice and control, and that their healthcare needs were respected. This was more frequently demonstrated in studies where women were seen by the same healthcare provider throughout their maternity care journey or when providers had a dedicated commitment to refugee health and caring for refugees.

*They have also picked up a couple of words in our language. They have been listening. It’s good cultural understanding you know. They ask you what you want to wear during labour*,* and I think we get validated without discrimination of our culture. (Woman from Somalia* [47]*).*

While many health professionals recognised the importance of providing continuity of care and culturally responsive care by the same provider, implementing these approaches in mainstream healthcare settings was challenging as it was often hindered by organisation and system-level factors outside their control.

*Each time you have to restart again. And you have to tell the same story*,* you have to. and yeah that kind of creates a kind of*,* yeah you don’t want to tell everything*,* because you need to*,* to get the relationship with your professional*,* and they don’t get it because you don’t see the same person each time. Each time you go there it’s someone different. (Refugee healthcare specialist* [17]*)*

In addition to the barriers experienced by healthcare providers identified previously, the workforce often faced moral and practical dilemmas when trying to adhere to organisational regulations while accommodating and respecting the women’s cultural needs. This further contributed to difficulties building trusting relationships. The barriers and enablers experienced by care providers were mapped against the ten strategies outlined in the EQUIP framework used to guide organisations in enhancing capacity for equity-oriented services. There was alignment in five strategies: (1) develop supportive organisational structures, policies, and procedures; (2) revision the use of time; (3) attend to power differentials; (4) tailor care, programs and services to local contexts; and (5) enhance access to the social determinants of health (Appendix F).

While most women who accessed mainstream maternity services were generally satisfied with the physical care received and reported that these needs were met, the psychosocial and practical needs of women were often overlooked or neglected by providers and services. Access to social support – both within and beyond the maternity care system – play a vital role in supporting women as they navigate pregnancy, childbirth, and early parenting during settlement in a new country. Women experienced social isolation and loneliness during pregnancy, childbirth, and the postpartum period, particularly after settling in a new country. The loss of traditional support systems – predominantly family members and female kin – was deeply felt and longed for by the women, especially during childbirth and the early postpartum period. Feelings of social isolation and loneliness were exacerbated during the postpartum period and were further heightened during the COVID-19 pandemic. Women valued the practical and psychosocial support they received from refugee-specific organisations, community members, and non-medical staff, particularly doulas and bicultural workers that shared their language, cultural, and religious backgrounds and migration journeys.

*‘…the doula was present*,* and it seemed like mum was present. She took care of me*,* showed love*,* and we need love. And it makes it easier to cope with the pain when you feel safe’ (Woman* [38]*).*

*Having people who can translate the culture…*,* the doulas and translators as part of our practice help[s] us. They give us tools.’’ (Medical Provider* [61]*)*

The significant role played by community-based maternity care staff was recognised as pivotal to supporting and improving maternity care experiences for women and supporting healthcare professionals in their work.

Women that participated in group pregnancy education classes also gained social and emotional support and connectedness from other mothers and were able to form friendships, share stories and experiences with them. These peer connections helped women to rebuild a sense of community and belonging and navigate motherhood in a new and unfamiliar environment.

#### Women’s settlement experiences influence their transition to new healthcare systems

This synthesised finding was generated through the aggregation of three categories, comprising 32 findings. When caring for women of refugee background, it is important to take a holistic approach that considers their pre-migration experiences, cultural and religious beliefs, and the challenges they face during settlement. These factors shape their health-seeking behaviour, access to care, and how their maternity care needs and preferences are understood and approached. Women’s pre-migration experiences included their histories of trauma, experiences of healthcare systems in their home countries, and traditional cultural and religious beliefs and norms around pregnancy, labour and birth, and the postpartum period. For many women, seeking care from hospitals or health services was associated with illness. Pregnancy and childbirth were viewed as natural life events that did not warrant additional medical care or intervention. This often led to hesitancy and mistrust in engaging with maternity care systems in countries of settlement, where pregnancy and birth are often medicalised and obstetric interventions are normalised.

*It is part of culture. Our women don’t like C-sections*,* they stay at home. The Somali women are scared to have the procedure. They stay at home longer and go through pain after pain after pain until she has no choice but to go to the hospital. (Woman from Somalia* [58]*)*

*I did not like it (pelvic exams). I feel it is not good to keep checking down there so I stopped going for appointments. (Woman from Somalia* [42]*)*.

Women were also apprehensive about discussing their postpartum mental health concerns with healthcare professionals due to the cultural stigma surrounding mental health illnesses. Many women feared that disclosing this sensitive information could result in women being seen as unfit or incapable of caring for their children by the health professionals.

For many, childbirth and the postpartum period are deeply meaningful and sacred life transitions. When Western models of care conflict with traditional cultural practices, this can leave women feeling that their cultural beliefs are not acknowledged or respected by healthcare professionals. Moreover, being separated from familiar cultural practices and support structures during this time can profoundly impact women’s experience of having a baby in a new country.

Women experienced a variety of challenges after arriving in a new country. These challenges included learning a new language, adjusting to foreign climates, navigating new systems such as healthcare, and coping with social isolation and the absence of familiar support systems. For many women, these challenges were compounded by the demands of pregnancy, caring for a newborn, or looking after other children. Balancing the stress of settlement, and roles as mothers and wives created an additional layer of stress of women, making it more difficult for them to adjust to life in a new country.

In their countries of settlement, many women experienced a shift in traditional gender roles during pregnancy, childbirth, and the postpartum period. Whilst female relatives usually accompanied and provided support to birthing and postpartum mothers in their home countries, husbands were often present during labour and childbirth in the countries of settlement and took on a more active role in the postpartum period caring for the women, in the absence of female support systems. This practice may have been considered unusual in the women’s home countries; however, it was welcomed by women and some of their husbands. Women appreciated the increased involvement of their husbands throughout pregnancy, childbirth, and postpartum.

*In my country father can’t come to delivery room. But I don’t know why*,* I think it’s very good*,* I like father being there*,* and my husband come to delivery room*,* it’s good for me*,* very good*,*.he’s always talk about delivery room*,* and he has good memories about see baby coming (laughs) (Woman from Afghanistan* [48]*)*

*“…[I] like Australia because the responsibility of raising children is shared [with husbands] and fathers are expected to do as much as mothers…” (Focus group participant)* [92]*)*.

Women also expressed a desire to build a future in their new country and wanted to support other women in the future to navigate the maternity care system.

## Discussion

This review synthesised the maternity care needs of women of refugee background settled in high-income countries and the barriers and enablers encountered by health, social, and community-based maternity care professionals in delivering such care, using qualitative evidence.

Our findings identified that women of refugee background value care delivered by the same (or a small group of known and trusted) female health, social, and community-based care professionals throughout their maternity care journey with care that is culturally responsive, trauma-informed, and woman- and family-centred. They also need services that are accessible, flexible, antenatal and postnatal health information translated in their preferred language resources, and access to social, psychological and practical support both within and beyond the maternity care system. The needs of women are shaped by both their pre-migration experiences (e.g., exposure to war and conflict, and traditional cultural and religious birth and postpartum practices) and post-settlement experiences, including challenges associated with settling in a new country and navigating structural and systemic barriers. Recognising these intersecting influences is critical for the provision of responsive and equitable care.

The needs of women in this review are consistent with the findings from existing literature on best practice models of maternity care for women of refugee background, such as those described by Correa-Velez and Ryan [93] Rogers et al. [94] and Fair et al. [20] However, our review extends prior work by applying a health and social equity lens, and mapping the maternity care needs of women and the barriers and enablers experienced by health and social care professionals who care for these women against an existing equity-oriented care framework (EQUIP) [35], an angle not previously explored in other systematic reviews. In applying the EQUIP framework to the findings of this review, it became clear that while the framework provided a valuable foundation, several additional dimensions were necessary to address the unique needs of women of refugee background. These included woman- and family-centred care, continuity of care, and continuity of carer, which reflect the central role of trust, relationships between woman and care provider, and family involvement in shaping positive maternity care experiences.

Trauma-informed care, culturally responsive care, woman- and family-centred care, and continuity of care and carer are key components to the provision of equity-oriented care during pregnancy, childbirth, and the postpartum period. While these approaches share overlapping principles, they are distinct to each other and are often implemented in isolation to each other in health and social equity interventions. The incorporation of trauma-informed care, culturally responsive care, woman and family-centred care, and continuity of care and carer in maternity models of care have shown to be beneficial in improving care experiences for women of refugee background [17, 40, 46, 47, 91, 95]. 

Trauma-informed care acknowledges the widespread impact of trauma and prioritises creating safe and trusting environments that minimise re-traumatisation for people impacted by trauma [96]. Providing culturally responsive care involves being respectful of the health beliefs, health practices, cultural and linguistic needs of diverse populations and communities [97]. Woman- and family-centred care prioritises women’s autonomy and agency throughout the childbearing experience, fostering relationships between women and care provider that are grounded in mutual trust and respect. This approach recognises and values women’s experiential wisdom and knowledge, and their capacity to meet their own individual needs. There is mutual understanding and decision-making between women and service providers, underpinned by evidence-based knowledge and practices. Through this relational approach, women are supported by their care providers to navigate complex health systems, ultimately resulting in improved health and wellbeing outcomes for both mother and child [98]. Continuity of care refers to where “a woman is cared for by a group of professionals with shared ways of working and a common philosophy” [99]. Continuity of carer means that a woman is being cared for by the same trusted carer throughout her maternity care journey [99]. 

When these approaches are brought together, they create a conceptual framework for improving women’s satisfaction with care, maternal health outcomes and health and social equity for women of refugee backgrounds and their families. When women are identified as of refugee background, maternity services should prioritise continuity of care models with trusted female providers and ensure more female professional interpreters are available and proactively offered throughout pregnancy, childbirth, and the postpartum period. The ascertainment of refugee background is critical for the provision of responsive, tailored and equitable care; however, accurate identification is challenging for clinicians as it requires approaching the subject sensitively. Many health professionals are insufficiently prepared or lack the necessary skills and confidence to undertake this task [100]. This challenge was evident in our review and is consistently reported in the wider literature. Guidance and professional development, informed by expert stakeholders with knowledge and lived experience, are needed to ensure that questions related to identifying refugee background are acceptable to women, clinicians and all involved [100]. 

This review also synthesised the main barriers and enablers experienced by health, social, and community-based care professionals working in maternity care services. Health, social, and community-based maternity care professionals reported barriers such as limited time, resources, and capacity to deliver adequate care and health information. Experiences of burnout, compassion fatigue and vicarious trauma were also identified among care providers, causing poor mental health and can reduce the quality of care provided [101]. Many health professionals working in mainstream organisations also lacked awareness and understanding of the cultural beliefs, needs, and customs of women, as well as knowledge of the context and experiences of people from refugee and asylum seeker backgrounds. This gap was largely attributed to limited professional development in cultural safety, refugee health, and trauma-informed approaches. Language and communication barriers – particularly the lack of access to professional interpreters during appointments – were also widely reported. Such barriers are consistently identified in the broader literature on care for migrants, refugees, and asylum seekers in high-income countries [30, 102]. 

Staff found it difficult building trusting relationships with the women they cared for, due to the communication barriers and the moral and practical dilemmas they encountered when trying to adhere to organisational policies while respecting women’s cultural needs. While the use of professional interpreters is necessary for bridging language gaps in the absence of a shared language, effective cross-cultural communication in health services extends well beyond interpreter use [14]. It requires clinicians to practice cultural sensitivity and cultural safety, and to integrate an understanding of trauma to provide responsive and tailored care that meets the needs of the women they care for [14]. To support this, mainstream maternity services with high refugee populations should mandate professional development in cultural safety, refugee health, and trauma-informed care for all staff [20]. Additionally, to support care provider mental health and wellbeing, psychological support should be offered to all staff who provide care to refugee background communities.

The presence of community-based maternity care providers such as bicultural workers and doulas, emerged as a key enabler that bridged the gaps between women and clinicians [39, 50, 91, 94]. The growth of this workforce in mainstream health services is greatly needed and requires the establishment of sustainable funding mechanisms to ensure their long-term integration. As part of the EQUIP framework, there are 10 strategies to guide organisations in enhancing capacity for equity-oriented services. We mapped the barriers and enablers experienced by care providers against these strategies, which aligned with half of the strategies: (1) develop supportive organisational structures, policies, and procedures; (2) revision the use of time; (3) attend to power differentials; (4) tailor care, programs and services to local contexts; and (5) enhance access to the social determinants of health (Table 2). This reinforces that the challenges faced by health professionals caring for women of refugee background largely stems from organisational, structural, and systemic barriers. While the strategies highlight key areas for equity-oriented organisational changes, broader system and structural reforms within the maternity care sector in high-income countries are crucial to achieving equitable maternity care for women of refugee background.

**Table 2:** Mapping the barriers and enablers experienced by health and social care professionals against the EQUIP framework

10 strategies to guide organizations in enhancing capacity for equity-oriented services	Description[104]	Enablers and barriers identified from review	
		Barriers	Enablers
Explicitly commit to equity	A strategic priority of the organisation and leadership is committed to improving equity at all levels of the organisation	N/A	N/A
Develop supportive organizational structures, policies, and processes	Structures, policies, and processes related to hiring, performance evaluation, recognition, rewards and compensation, continuing education, and staff meetings all are viewed with respect to equity. For example, staff whose values align with the commitment to equity are recruited, hired and retained. There are also dedicated resources in the budget to support equity work.	• Moral and practical dilemmas fuelled by organisational regulations • Lack of resources • Insufficient specialised training • Lack of appropriate skills	Social support staff who supported healthcare professionals in their work
Revision the use of time	For example, flexibility with scheduling appointments recognizing patients facing structural vulnerabilities may not arrive on time, or keep scheduled appointments, or may only seek emergency care. How well does the practice accommodate patients seeking more time for procedures? How well are the tensions between the need to produce (efficiencies) and the needs of priority patient groups (appropriateness) planned and accounted for?	• Limited time • Lack of capacity	N/A
Attend to power differentials	All staff having some influence on how the organization’s work activities are carried out. During interactions with patients, this means paying attention to how you might be perceived as intimidating to patients, regardless of your intention or actions that are aimed at making patients feel comfortable and welcome.	Moral and practical dilemmas fuelled by organisational regulations contributing to difficulties building trusting relationships	N/A
Tailor care, programs and services to local contexts	Context refers to the broader cultures, structures, political systems, and local communities within a particular place. It is important to know and understand these to effectively tailor services to local contexts. How well has the organization tailored care to uniquely address the known barriers to access care for underserved populations? How distinct is the organization from mainstream practices? For example, how well are financial barriers addressed for people without benefits or with public plans and experiencing poverty?	• Lack of access to professional interpreters (communication challenges) • Lack of cultural awareness (cultural differences in care) • Lack of knowledge	Diverse and innovative communication strategies
Actively counter racism and discrimination	For example, staff members actively respond to discriminatory comments when they encounter them. Claims of discrimination are also considered seriously, regardless of intention. Practice is free of discrimination and differential treatment based on income level and source of income and health benefits. Practice is free of discrimination based on social location, life histories and health challenges such as substance use, experiencing homelessness, poor hygiene or less-typical appearance or behaviours	N/A	N/A
Promote meaningful community and patient engagement	Patients and community members have an active voice in their care and are encouraged to provide feedback to the organization. Does the organization have deliberate practices to engage patients in planning processes or through a patient committee?	N/A	N/A
Tailor care to address inter-related forms of violence	Some people may be survivors of multiple forms of violence with traumatic effects, while still experiencing current and ongoing interpersonal violence (including racial violence and intimate partner violence), and ongoing structural violence (such as systemic and organizational racism, absolute poverty, etc.). How well is this reflected in the care and services provided? What is the history with First Nations peoples in that area? How well does your organization know the local context? How well is that reflected in the care and services provided?	N/A	N/A
Enhance access to the social determinants of health	Some aspects of peoples’ everyday lives that have major impacts on health, for example, access to affordable, safe housing, income level above the poverty line (social assistance/disability incomes are not), and interactions in the social world that are respectful, non-stigmatizing, and non-discriminatory. How well do payment policies and practices respond to economic vulnerabilities and the limitations of public policy plans? How are the determinants of health acknowledged as part of treatment and prevention? How are Electronic Medical Records used to document the social determinants of health?	National housing and migration policies	N/A
Optimise use of place and space	What messages are reflected in the way the space is designed? Is the space designed to be inclusive of those who typically are marginalized? Would people from priority populations see themselves reflected in the design of the space? Are services located in the neighbourhoods where people who are underserved may likely reside? Are transportation issues (including cost) considered?	N/A	N/A

Future research should focus on the co-design of tailored equity-oriented maternity care interventions that integrate trauma-informed care, culturally responsive care, woman and family-centred care, and continuity of care and carer with maternity services and trusted community organisations. Furthermore, evaluation of these programs should be undertaken to determine their impact on participants, their families and health professionals.

### Strengths and limitations

A major strength of this review is that it is the first comprehensive systematic review of qualitative evidence to synthesise *both* the maternity care needs of women of refugee background living in high-income countries and the barriers and enablers experienced by health, social, and community-based maternity care professionals when delivering care, within a health and social equity lens. This unique review offers a comprehensive and nuanced understanding of both perspectives in a single review, contributing to a gap in existing literature. While synthesising both women and providers’ perspectives limited the depth of analysis within each group, this integrative approach enabled the examination of relational, structural and systemic interactions that is absent in single-perspective reviews.

A further strength of this review is that it highlights the needs of women of refugee background, ensuring recommendations are tailored to specifically address the unique needs of this population. There is limited evidence on these types of studies as most studies group the experiences of refugee women together with other migrant groups.

A final strength is the geographic variety of high-income nations from Europe, Australia and North America represented in this review, enhancing the breadth and representativeness of the findings. While structural funding arrangements differ across high-income countries, we found themes of shared experience of forced displacement and common barriers to interpreter access, continuity of care, and culturally responsive care were present across studies, suggesting that these findings reflect recurring systemic patterns rather than isolated national issues. Readers are encouraged to consider the context of their own specific health system when drawing on the findings of this review.

This review has several limitations which need to be acknowledged. Firstly, studies were only included if they were published in English which may have excluded relevant studies published in other languages. Another limitation was that while we tried our best to only include studies where the study population was reported as ‘refugee’, many studies had ambiguous descriptions of refugee populations, poorly defined their study population, or used terminologies such as ‘refugee’ or ‘asylum seeker’ interchangeably with ‘migrant’, which may have resulted in the exclusion of these papers during the screening process despite a comprehensive and rigorous search approach. Finally, while we explicitly excluded studies where the study population were trafficked women or undocumented migrants, we acknowledge that forced displacement, trafficking and undocumented status are not always mutually exclusive experiences, and that some participants of refugee background may have also had experience of trafficking or periods of undocumented status. However, this could not be clearly identifiable from the available information.

### Reflexivity

The review team comprised five female qualitative researchers with a shared commitment to health and social equity and equitable maternity care for women of refugee background. The team held expertise in maternal and child health, public health, refugee and migrant health, and qualitative systematic review methods (specifically JBI). We brought our diverse perspectives and positionalities to the review, including migrant backgrounds, clinical expertise (obstetrics/gynaecology, midwifery, and paediatric nursing), and personal experiences of receiving maternity care in Australia. These disciplinary, cultural, and experiential lenses inevitably shaped how we engaged with and interpreted the data. Throughout the review process, we engaged in regular reflexive dialogue, particularly during screening, synthesis and writing, to critically examine how our disciplinary lenses and lived experiences influenced the coding and categorisation of data. Rather than treating differences in interpretation as discrepancies to be resolved, we viewed them as opportunities to interrogate the findings more deeply, thereby enriching the analysis.

## Conclusions

The maternity care needs of women of refugee background settled in high-income countries remain neglected and unmet, contributing to persistent inequities in experiences of care and health outcomes for these women and their babies. This review has synthesised qualitative evidence on the maternity care needs of women of refugee background and the barriers and enablers for providing care to this population, within a health and social equity lens. Three main findings were identified: (1) Structural and systemic factors shape the provision and access to equity-oriented maternity care; (2) Key components to equity-oriented care are: trauma-informed care, culturally responsive care, woman- and family-centred care, and continuity of care and carer and (3) Women’s settlement experiences influence their transition to new healthcare systems. To address the persistent health inequities experienced by women of refugee background in high-income countries and achieve equitable maternity care, systemic reform, sustainable investments in the workforce, specialised training for care providers, and further research on the co-design and evaluation of equity-oriented care interventions is required in maternity services to better respond to the needs of this population.

## Supplementary Information

Below is the link to the electronic supplementary material.

Supplementary Material 1

## Data Availability

All relevant data are included in the article and its supporting information file.
