# Supplementary material for: Understanding equity-oriented maternity care for women of refugee background in high-income countries: a qualitative systematic review
Source: Int J Equity Health. 2026 Apr 20;25:106. doi: 10.1186/s12939-026-02848-5 (PMC13097718; doi:10.1186/s12939-026-02848-5)
Supplement: Supplementary file 1 — Supplementary Material 1 [file 12939_2026_2848_MOESM1_ESM.docx]

# Supplementary Information

## Appendix A: Search strategy

### CINAHL

| S23 | S3 AND S12 AND S16 AND S22 | **Limiters** - Publication Date: 20130101-; Peer Reviewed  **Search modes** - Boolean/Phrase |
| --- | --- | --- |
|  | S22 | S17 OR S18 OR S19 OR S20 OR S21 |
|  | S21 | cultural* or equity or equitable or trauma-informed or violence-informed-care or anti-racist or antiracist or anti-discriminatory or respectful or person-centred or person-centered or access* or women-centred or women-centered or woman-centred or woman-centered or family-centered or family-centred |
|  | S20 | (MH "Patient Centered Care") |
|  | S19 | (MH "Diversity, Equity, Inclusion") |
|  | S18 | (MH "Health Care Delivery") OR (MH "Health Services Accessibility+") OR (MH "Primary Health Care") OR (MH "Healthcare Disparities") |
|  | S17 | (MH "Cultural Competence") OR (MH "Transcultural Care") |
|  | S16 | S13 OR S14 OR S15 |
|  | S15 | (MH "Developed Countries") |
|  | S14 | developed-countr* or developed-nation* or industrialized-countr* or industrialized-nation* or industrialised-countr* or industrialised-nation* |
|  | S13 | TX Norway or Switzerland or Ireland or Hong-Kong or Iceland or Germany or Sweden or Australia or Netherlands or Denmark or Singapore or Finland or United-Kingdom or New-Zealand or Belgium or Canada or United-States or Austria or Liechtenstein or Japan or Israel or Slovenia or Luxembourg or South-Korea or Andorra or Latvia or Portugal or Slovakia or Spain or France or Czech* or Malta or italy or Estonia or United-Arab-Emirates or Greece or Cyprus or Lithuania or Poland or UK or US or USA or UAE o [...](javascript:showHistoryTerm('ctl00_ctl00_MainContentArea_MainContentArea_historyControl_HistoryRepeater_ctl10_ellipsis',true)) |
|  | S12 | S4 OR S5 OR S6 OR S7 OR S8 OR S9 OR S10 OR S11 |
|  | S11 | parturition or ante-natal or antenatal* or pre-natal* or prenatal* or puerper* or postnatal* or post-natal* or postpartum or post-partum or peripartum or peri-partum or periconception* or peri-conception* or ((preterm or prematur*) and (labor or labour)) or breastfe* or breast-fe* or lactation* or cesarean or caesarean or cesarian or caesarian or cesarien or caesarien or tocoly* or fetal or foetal or fetus or foetus or miscarriage* or pregnancy or pregnancies or pregnant or perinatal or peri-nat [...](javascript:showHistoryTerm('ctl00_ctl00_MainContentArea_MainContentArea_historyControl_HistoryRepeater_ctl12_ellipsis',true)) |
|  | S10 | (MH "Maternal Health Services+") |
|  | S9 | (MH "Labor Pain") |
|  | S8 | (MH "Childbirth Education") |
|  | S7 | (MH "Breast Feeding+") |
|  | S6 | (MH "Obstetrics") |
|  | S5 | (MH "Pregnancy+") |
|  | S4 | (MH "Expectant Mothers") |
|  | S3 | S1 OR S2 |
|  | S2 | asylum or refugee* or alien or aliens or crisis-affected-population* or displaced-people or displaced-person* or forced-displacement* or forced-migra* or involuntary-migra* or involuntary-immigra* or humanitarian-entrant* |
|  | S1 | (MH "Refugees") |

### Embase (OVID)

| 1. | exp forced migrant/ |
| --- | --- |
| 2. | (asylum or refugee* or alien? or crisis-affected-population* or displaced-people or displaced-person* or forced-displacement* or forced-migra* or involuntary-migra* or involuntary-immigra* or humanitarian-entrant*).tw,kf,dq. |
| 3. | 1 or 2 |
| 4. | exp PREGNANCY/ or exp OBSTETRIC PROCEDURE/ or exp BREAST FEEDING/ or exp BREAST FEEDING EDUCATION/ or exp BIRTH/ or exp CHILDBIRTH/ or CHILDBIRTH EDUCATION/ or LABOR PAIN/ or maternal health service/ or (parturition or ante-natal or antenatal* or pre-natal* or prenatal* or puerper* or postnatal* or post-natal* or postpartum or post-partum or peripartum or peri-partum or periconception* or peri-conception* or ((preterm or prematur*) and (labor or labour)) or breastfe* or breast-fe* or lactation* or cesarean or caesarean or cesarian or caesarian or cesarien or caesarien or tocoly* or fetal or foetal or fetus or foetus or miscarriage* or pregnancy or pregnancies or pregnant or perinatal or peri-natal or mother or mothers or matern* or obstetric* or labor-pain* or labour-pain* or childbirth or birth* or midwife* or midwives).tw,kf,dq. |
| 5. | (Norway or Switzerland or Ireland or Hong-Kong or Iceland or Germany or Sweden or Australia or Netherlands or Denmark or Singapore or Finland or United-Kingdom or New-Zealand or Belgium or Canada or United-States or Austria or Liechtenstein or Japan or Israel or Slovenia or Luxembourg or South-Korea or Andorra or Latvia or Portugal or Slovakia or Spain or France or Czech* or Malta or italy or Estonia or United-Arab-Emirates or Greece or Cyprus or Lithuania or Poland or UK or US or USA or UAE or NZ or Greenland or United-States or Hong-Kong or HK or Croatia).tw,kf,dq,in. |
| 6. | exp canada/ or exp united states/ or Singapore/ or Israel/ or exp United Arab Emirates/ or hong kong/ or Japan/ or South Korea/ or Andorra/ or austria/ or exp belgium/ or exp Baltic States/ or croatia/ or czech republic/ or poland/ or slovakia/ or slovenia/ or exp france/ or exp germany/ or exp united kingdom/ or greece/ or ireland/ or exp italy/ or liechtenstein/ or luxembourg/ or Cyprus/ or malta/ or netherlands/ or exp portugal/ or exp Scandinavia/ or exp spain/ or switzerland/ or exp australia/ or new zealand/ |
| 7. | (developed-countr* or developed-nation* or industrialized-countr* or industrialized-nation* or industrialised-countr* or industrialised-nation*).tw,kf,dq. |
| 8. | developed country/ |
| 9. | 5 or 6 or 7 or 8 |
| 10. | transcultural care/ |
| 11. | cultural competence/ |
| 12. | health care delivery/ or exp health care access/ or exp primary health care/ |
| 13. | health equity/ |
| 14. | patient satisfaction/ |
| 15. | patient care/ or cultural safety/ or exp holistic care/ |
| 16. | (cultural* or equity or equitable or trauma-informed or violence-informed-care or anti-racist or antiracist or anti-discriminatory or respectful or person-centred or person-centered or access* or wom#n-centred or wom#n-centered or family-centered or family-centred).tw,kf,dq. |
| 17. | 10 or 11 or 12 or 13 or 14 or 15 or 16 |
| 18. | 3 and 4 and 9 and 17 |
| 19. | limit 18 to yr="2013 -Current" |
| 20. | case report/ |
| 21. | limit 19 to (conference abstract or conference paper or "conference review" or editorial or letter or "preprint (unpublished, non-peer reviewed)") |
| 22. | 19 not (20 or 21) |

### Medline (OVID)

| 1. | *refugees/ |
| --- | --- |
| 2. | (asylum or refugee* or alien? or crisis-affected-population* or displaced-people or displaced-person* or forced-displacement* or forced-migra* or involuntary-migra* or involuntary-immigra* or humanitarian-entrant*).tw,kf. |
| 3. | 1 or 2 |
| 4. | exp *pregnant women/ or exp *Pregnancy/ or exp *Obstetrics/ or exp *Breast Feeding/ or exp *Prenatal Education/ or exp *Labor Pain/ or exp *Maternal Health Services/ or (parturition or ante-natal or antenatal* or pre-natal* or prenatal* or puerper* or postnatal* or post-natal* or postpartum or post-partum or peripartum or peri-partum or periconception* or peri-conception* or ((preterm or prematur*) and (labor or labour)) or breastfe* or breast-fe* or lactation* or cesarean or caesarean or cesarian or caesarian or cesarien or caesarien or tocoly* or fetal or foetal or fetus or foetus or miscarriage* or pregnancy or pregnancies or pregnant or perinatal or peri-natal or mother or mothers or matern* or obstetric* or labor-pain* or labour-pain* or childbirth or birth* or midwife* or midwives).tw,kf. |
| 5. | (Norway or Switzerland or Ireland or Hong-Kong or Iceland or Germany or Sweden or Australia or Netherlands or Denmark or Singapore or Finland or United-Kingdom or New-Zealand or Belgium or Canada or United-States or Austria or Liechtenstein or Japan or Israel or Slovenia or Luxembourg or South-Korea or Andorra or Latvia or Portugal or Slovakia or Spain or France or Czech* or Malta or Italy or Estonia or United-Arab-Emirates or Greece or Cyprus or Lithuania or Poland or UK or US or USA or UAE or NZ or Greenland or United-States or Hong-Kong or HK or Croatia).tw,kf,in. |
| 6. | exp canada/ or greenland/ or exp united states/ or singapore/ or israel/ or united arab emirates/ or hong kong/ or exp japan/ or exp "republic of korea"/ or andorra/ or austria/ or belgium/ or exp baltic states/ or croatia/ or czech republic/ or poland/ or slovakia/ or slovenia/ or exp france/ or exp germany/ or exp united kingdom/ or greece/ or ireland/ or italy/ or liechtenstein/ or luxembourg/ or cyprus/ or malta/ or netherlands/ or portugal/ or exp "scandinavian and nordic countries"/ or spain/ or switzerland/ or exp australia/ or iceland/ or japan/ or new zealand/ |
| 7. | (developed-countr* or developed-nation* or industrialized-countr* or industrialized-nation* or industrialised-countr* or industrialised-nation*).tw,kf. |
| 8. | Developed Countries/ |
| 9. | 5 or 6 or 7 or 8 |
| 10. | Culturally Competent Care/ or Cultural Competency/ |
| 11. | "delivery of health care"/ or health services accessibility/ or access to primary care/ or health equity/ or exp Patient Satisfaction/ |
| 12. | Patient-Centered Care/ |
| 13. | (cultural* or equity or equitable or trauma-informed or violence-informed-care or anti-racist or antiracist or anti-discriminatory or respectful or person-centred or person-centered or access* or wom#n-centred or wom#n-centered or family-centered or family-centred).tw,kf. |
| 14. | 10 or 11 or 12 or 13 |
| 15. | 3 and 4 and 9 and 14 |
| 16. | limit 15 to yr="2013 -Current" |
| 17. | limit 16 to (case reports or comment or editorial or guideline or letter or practice guideline or preprint) |
| 18. | 16 not 17 |

### PubMed

#1 title/abstract

“asylum” OR “refugee*” OR “alien” OR “aliens” OR “crisis-affected-population*” OR “displaced-people” OR “displaced-person*” OR “forced-displacement*” OR “forced-migra*” OR “involuntary-migra*” OR “involuntary-immigra*” OR “humanitarian-entrant*”

#2 title/abstract

“Obstetric*” OR “parturition” OR “ante-natal” OR “antenatal*” OR “pre-natal*” OR “prenatal*” OR “puerper*” OR “postnatal*” OR “post-natal*” OR “postpartum” OR “post-partum” OR “peripartum” OR “peri-partum” OR “periconception*” OR “peri-conception*” OR “breastfe*” OR “breast-fe*” OR “lactation*” OR “cesarean” OR “caesarean” OR “cesarian” OR “caesarian” OR “cesarien” OR “caesarien” OR “tocoly*” OR “fetal” OR “foetal” OR “fetus” OR “foetus” OR “miscarriage*” OR “pregnancy” OR “pregnancies” OR “pregnant” OR “perinatal” OR “peri-natal” OR “mother” OR “mothers” OR “matern*” OR “obstetric*” OR “labor-pain*” OR “labour-pain*” OR “childbirth” OR “birth*” OR “midwife*” OR “midwives”

#3 title/abstract

(“preterm” OR “prematur*”) AND (“labor” OR “labour”)

#4 #2 OR #3

#5 title/abstract

“Norway” OR “Switzerland” OR “Ireland” OR “Hong-Kong” OR “Iceland” OR “Germany” OR “Sweden” OR “Australia” OR “Netherlands” OR “Denmark” OR “Singapore” OR “Finland” OR “United-Kingdom” OR “New-Zealand” OR “Belgium” OR “Canada” OR “United-States” OR “Austria” OR “Liechtenstein” OR “Japan” OR “Israel” OR “Slovenia” OR “Luxembourg” OR “South-Korea” OR “Andorra” OR “Latvia” OR “Portugal” OR “Slovakia” OR “Spain” OR “France” OR “Czech*” OR “Malta” OR “Italy” OR “Estonia” OR “United-Arab-Emirates” OR “Greece” OR “Cyprus” OR “Lithuania” OR “Poland” OR “UK” OR “US” OR “USA” OR “UAE” OR “NZ” OR “Greenland” OR “United-States” OR “Hong-Kong” OR “HK” OR “Croatia” OR “developed-countr*” OR “developed-nation*” OR “industrialized-countr*” OR “industrialized-nation*”

#6 title/abstract

“cultural*” OR “transcultural” OR “equity” OR “equitable” OR “trauma-informed” OR “violence-informed-care” OR “anti-racist” OR “antiracist” OR “anti-discriminatory” OR “respectful” OR “person-centred” OR “person-centered” OR “access*” OR “women-centred” OR “women-centered” OR “woman-centred” OR “woman-centered” OR “family-centered” OR “family-centred” OR “patient-centered” OR “patient-centred” OR “holistic” OR “delivery-of-health*” OR “access*” OR “satisfaction”

#7 all fields

NOTNLM OR publisher[sb] OR inprocess[sb] OR pubmednotmedline[sb] OR indatareview[sb] OR pubstatusaheadofprint

#8 #1 AND #4 AND #5 AND #6 AND #7

### Web of Science

#1

“asylum” OR “refugee*” OR “alien” OR “aliens” OR “crisis-affected-population*” OR “displaced-people” OR “displaced-person*” OR “forced-displacement*” OR “forced-migra*” OR “involuntary-migra*” OR “involuntary-immigra*” OR “humanitarian-entrant*”

#2

“Obstetric*” OR “parturition” OR “ante-natal” OR “antenatal*” OR “pre-natal” OR “prenatal” OR “puerper*” OR “postnatal*” OR “post-natal*” OR “postpartum” OR “post-partum” OR “peripartum” OR “peri-partum” OR “periconception*” OR “peri-conception*” OR “breastfe*” OR “breast-fe*” OR “lactation*” OR “cesarean” OR “caesarean” OR “cesarian” OR “caesarian” OR “cesarien” OR “caesarien” OR “tocoly*” OR “fetal” OR “foetal” OR “fetus” OR “foetus” OR “miscarriage*” OR “pregnancy” OR “pregnancies” OR “pregnant” OR “perinatal” OR “peri-natal” OR “mother” OR “mothers” OR “matern*” OR “obstetric*” OR “labor-pain*” OR “labour-pain*” OR “childbirth” OR “birth*” OR “midwife*” OR “midwives”

#3

(“preterm” OR “prematur*”) AND (“labor” OR “labour”)

#4 #2 OR #3

#5

“Norway” OR “Switzerland” OR “Ireland” OR “Hong-Kong” OR “Iceland” OR “Germany” OR “Sweden” OR “Australia” OR “Netherlands” OR “Denmark” OR “Singapore” OR “Finland” OR “United-Kingdom” OR “New-Zealand” OR “Belgium” OR “Canada” OR “United-States” OR “Austria” OR “Liechtenstein” OR “Japan” OR “Israel” OR “Slovenia” OR “Luxembourg” OR “South-Korea” OR “Andorra” OR “Latvia” OR “Portugal” OR “Slovakia” OR “Spain” OR “France” OR “Czech*” OR “Malta” OR “Italy” OR “Estonia” OR “United-Arab-Emirates” OR “Greece” OR “Cyprus” OR “Lithuania” OR “Poland” OR “UK” OR “US” OR “USA” OR “UAE” OR “NZ” OR “Greenland” OR “United-States” OR “Hong-Kong” OR “HK” OR “Croatia” OR “developed-countr*” OR “developed-nation*” OR “industrialized-countr*” OR “industrialized-nation*”

#6

“cultural*” OR “transcultural” OR “equity” OR “equitable” OR “trauma-informed” OR “violence-informed-care” OR “anti-racist” OR “antiracist” OR “anti-discriminatory” OR “respectful” OR “person-centred” OR “person-centered” OR “access*” OR “women-centred” OR “women-centered” OR “woman-centred” OR “woman-centered” OR “family-centered” OR “family-centred” OR “patient-centered” OR “patient-centred” OR “holistic” OR “delivery-of-health*” OR “access*” OR “satisfaction”

#7 #1 AND #4 AND #5 AND #6

## Appendix B: Assessment of methodological quality

| **Study** | **JBI Critical Appraisal Checklist** | | | | | | | | | |
| --- | --- | --- | --- | --- | --- | --- | --- | --- | --- | --- |
|  | **Q1** | **Q2** | **Q3** | **Q4** | **Q5** | **Q6** | **Q7** | **Q8** | **Q9** | **Q10** |
| Agbemenu et al. (2021) | U | Y | Y | Y | Y | Y | N | Y | Y | Y |
| Alsamman et al. (2025) | U | Y | Y | Y | Y | Y | N | Y | Y | Y |
| Asbjornsen et al. (2025) | Y | Y | Y | Y | Y | Y | Y | Y | Y | Y |
| Ayers et al. (2025) | U | Y | Y | Y | Y | N | N | Y | Y | Y |
| Banke-Thomas et al. (2017) |  |  |  |  |  |  |  |  |  |  |
| Cameron et al. (2021) | Y | Y | Y | Y | Y | N | N | Y | Y | Y |
| Cameron et al. (2022) | Y | Y | Y | Y | Y | N | N | Y | Y | Y |
| Chrzan-Dętkoś & Murawska  (2023) | U | Y | Y | Y | Y | N | N | Y | N | Y |
| Coe et al. (2024) | Y | Y | Y | Y | Y | Y | N | Y | Y | Y |
| Denzongpa & Nichols (2023) | Y | Y | Y | Y | Y | Y | Y | Y | Y | Y |
| Dube et al. (2024) | U | Y | Y | Y | Y | Y | Y | Y | Y | Y |
| Dube et al. (2025) | Y | Y | Y | Y | Y | Y | Y | Y | Y | Y |
| Due et al. (2022) | U | Y | Y | Y | Y | Y | Y | Y | Y | Y |
| Erga-Johansen & Bondas (2023) | Y | Y | Y | Y | Y | Y | Y | Y | Y | Y |
| Evans et al. (2022) | U | Y | Y | Y | Y | N | N | Y | Y | Y |
| Fair et al. (2021) | U | Y | Y | Y | Y | N | N | Y | Y | Y |
| Gateri (2024) | Y | Y | Y | Y | Y | Y | Y | Y | Y | Y |
| Glavin & Sæteren (2016) | U | Y | Y | Y | Y | N | Y | Y | Y | Y |
| Haith-Cooper & Bradshaw (2013) | U | Y | Y | Y | Y | N | N | Y | Y | Y |
| Hearn et al. (2023) | Y | Y | Y | Y | Y | Y | Y | Y | Y | Y |
| Hearn et al. (2024) | Y | Y | Y | Y | Y | Y | Y | Y | Y | Y |
| Henry et al. (2020) | Y | Y | Y | Y | Y | Y | Y | Y | Y | Y |
| Higginbottom et al. (2013) | U | Y | Y | Y | Y | N | N | Y | Y | Y |
| Khaw et al. (2023) | Y | Y | Y | Y | Y | Y | Y | Y | Y | Y |
| Kirkdendall & Dutt (2023) | Y | Y | Y | Y | Y | Y | Y | Y | Y | Y |
| LaMancuso et al. (2016) | Y | Y | Y | Y | Y | N | N | Y | Y | Y |
| Lazar et al. (2013) | U | Y | Y | Y | Y | N | Y | Y | Y | Y |
| Leoniuk et al. (2025) | U | Y | Y | Y | Y | N | N | Y | Y | Y |
| Lephard & Haith-Cooper (2016) | Y | Y | Y | Y | Y | N | N | Y | Y | Y |
| Lillrank (2015) | Y | Y | Y | Y | Y | N | Y | Y | Y | Y |
| Lukasse et al. (2025) | U | Y | Y | Y | Y | Y | Y | Y | Y | Y |
| Mendel et al. (2021) | Y | Y | Y | Y | Y | Y | Y | Y | Y | Y |
| Missal et al. (2016) | Y | Y | Y | Y | Y | N | Y | Y | Y | Y |
| Nenko et al. (2024) | U | Y | Y | Y | Y | N | Y | Y | Y | Y |
| Njenga (2022) | U | Y | Y | Y | Y | Y | N | Y | Y | Y |
| Njenga (2023) | Y | Y | Y | Y | Y | Y | N | Y | Y | Y |
| Olcoń et al. (2023) | U | Y | Y | Y | Y | N | N | N | Y | Y |
| Owens et al. (2016) | Y | Y | Y | Y | Y | Y | Y | Y | Y | Y |
| Papadakaki et al. (2021) | U | Y | Y | Y | Y | N | N | Y | Y | Y |
| Pierce et al. (2025) | U | Y | Y | Y | Y | N | N | Y | Y | Y |
| Riggs et al. (2017) | U | Y | Y | Y | Y | N | N | Y | Y | Y |
| Rogers et al. (2021) | U | Y | Y | Y | Y | N | Y | Y | Y | Y |
| Rowe et al. (2023) | U | Y | Y | Y | Y | N | Y | Y | Y | Y |
| Russo et al. (2015) | Y | Y | Y | Y | Y | Y | Y | Y | Y | Y |
| Smith et al. (2025) | Y | Y | Y | Y | Y | Y | Y | Y | Y | Y |
| Stapleton et al. (2013) | U | Y | Y | Y | Y | N | N | N | Y | Y |
| Tankink et al. (2024) | Y | Y | Y | Y | Y | Y | Y | Y | Y | Y |
| Tobin & Murphy-Lawless (2014) | U | Y | Y | Y | Y | N | N | N | Y | Y |
| Tobin et al. (2014) | Y | Y | Y | Y | Y | N | N | Y | Y | Y |
| Toke et al. (2024) | Y | Y | Y | Y | Y | Y | N | Y | Y | Y |
| Verschuuren et al. (2023) | U | Y | Y | Y | Y | N | N | Y | N | Y |
| Willey et al. (2018) | U | Y | Y | Y | Y | N | Y | Y | Y | Y |
| Winn et al. (2018) | Y | Y | Y | Y | Y | N | N | Y | Y | Y |
| Worabo et al. (2024) | U | Y | Y | Y | Y | Y | N | Y | Y | Y |
| Yelland et al. (2014) | U | Y | Y | Y | Y | N | N | Y | Y | Y |
| Yeo et al. (2024) | Y | Y | Y | Y | Y | N | N | Y | Y | Y |

Y=Yes; N=No; U=Unclear

Q1= Is there congruity between the stated philosophical perspective and the research methodology?

Q2= Is there congruity between the research methodology and the research question or objectives?

Q3= Is there congruity between the research methodology and the methods used to collect data?

Q4= Is there congruity between the research methodology and the representation and analysis of data?

Q5= Is there congruity between the research methodology and the interpretation of results?

Q6= Is there a statement locating the researcher culturally or theoretically?

Q7= Is the influence of the researcher on the research, and vice- versa, addressed?

Q8= Are participants, and their voices, adequately represented?

Q9= Is the research ethical according to current criteria or, for recent studies, and is there evidence of ethical approval by an appropriate body?

Q10= Do the conclusions drawn in the research report flow from the analysis, or interpretation, of the data?

## Appendix C: Findings and illustrations from each study

| **Study** | **Finding** | **Illustration** |
| --- | --- | --- |
| Agbemenu et al. (2021) | Intentionally not seeking or misleading prenatal care | Going for prenatal care, we are afraid, we just feel mistreated and disrespected by the exposure because we have seen it before, we know it [other people will be in the consulting room] will happen again. (M5F4) |
|  | Changing hospitals and/or providers when care has commenced | I went to midwives because they cannot perform C-sections…I went to them because I was running away from C-section. (M4F3) |
|  | Delayed hospital arrival during labour | It is part of culture. Our women don’t like C-sections, they stay at home. The Somali women are scared to have the procedure. They stay at home longer and go through pain after pain after pain until she has no choice but to go to the hospital. (M3F3) |
|  | Outright refusal of care | What can they do? They are tools in the hands of God. Only God knows, the predestination. Sometimes, they [health workers] do their own will…. I cannot trust such people [health workers]. When one doctor told me she will cut me, I refused the care. Even if I did not, my husband would have. We will trust in God, even if the baby dies in my womb. (M1F2) |
| Alsamman et al. (2025) | Lack of comprehensible postpartum information | “Yes, I still have questions about how and when to take the medication I was prescribed postpartum. I asked my young son to read the discharge papers for  me.” (Afghanistan, Age 30). |
|  | Displacement and isolation worsen postpartum mental health | “I cried every day. I am stressed. I am in a foreign country. I fled a country in the midst of war and there were bombs being dropped on us. I am worried about my newborn son. I am worried about my  daughters. I am worried about my life. This [emotional reaction] is something normal that people experience after going through all the things that I went through. My body has not even acclimated to the US, and so of course I had postpartum depression.”  (Syria, Age 41). |
|  | Stigma and fear discourage seeking postpartum mental health care | “I must be cautious of what I say about my feelings because I don’t want them to think I have any psychological problems. […] I had a lot of things going on postpartum—I was in pain, I was stressed, and I could not pray for 16 days. I felt like I was going to explode. I just wanted to cry, but I was afraid that they would take my son away from me. So, I did not talk about my feelings at all.” (Syria, Age 41). |
|  | Barriers in interpretation undermine postpartum care recommendations | “The hardest thing is the language barrier. Sometimes when I want to express how I feel, even though the interpreter understands me, they do not interpret  what I am saying correctly or do not get the right point across. Therefore, I find it very hard to get my point across to the doctors.” Syria, Age 41). |
|  | Interest in a language concordant postpartum navigator | “I think it would be useful to have a program like this, especially for inexperienced mothers like me. For instance, I did not know that I could visit  the doctor postpartum. This would help us a lot.” (Afghanistan, Age 28). |
| Asbjornsen et al. (2025) | The role of intersecting identities in shaping maternal healthcare needs | I was three months pregnant. I was so tired and exhausted. I just wanted to sleep. I did not manage to have control over my body. I was so tired. That is why I needed sick leave from the Introduction Course. It was just so heavy. Getting the children to school, getting myself ready, rushing home after classes, make dinner…So it was hard. (Participant 5). |
|  | Experiences of structural inequities in maternal healthcare services | If you do not have the language, you are unable to search online for information, travel to the healthcare facilities, call the midwife to get easier access to services. The knowledge the women have about the  system is often not because they asked health personnel, but because friends told them. I tell them that they need to ask the midwife because that information is often better. (Participant 7). |
|  | Adaptation and its influence on pregnancy, birth and postpartum experiences | I feel quite a mess. I have backache, many children at home, and soon I must start work practice. I miss my family. My father is ill. I haven’t seen them in 13 years. Things are not quite right…I have 6 children you know, and a lot of absences from the introduc­tion program. It causes a lot of problems for me. (Participant 2). |
| Ayers et al. (2025) | Experiences with immigrant clients | I work closely with our Marshallese clients; language barriers are for sure one of the big things. … Most of them are not aware of what’s going on. The doctors or nurses will usually do whatever they have to do with them. They [clients] just let it happen. Language barriers is one thing I see as a big struggle with my clients. They aren’t aware of what’s going on. (Doula 3) |
|  | Experiences with maternal health-care providers | One incidence that I did have was that I came in with [client] mom and she was already dilated. She had labored mostly at home because that was her choice to labor as much as she could at home. And when we got there it was such a rush. I get it, hospitals are a different world. I want to respect the people that are working, but I do think that sometimes people are overlooked. I think because there is so much going on they don’t take the time to actively listen to the mom. They were telling her not to lay the way she was laying but that was what was comfortable to her and that was what was going to allow her to be ready to push. We had to be assertive and say, “We know what we are doing.” (Doula 6) |
|  | Suggestions to improve support for immigrant clients | The doctor is not listening to them. For example, they might tell the doctor, “I have this pain between my hip and my belly.” And the doctor will say “Oh, that is normal.” A lot my girls understand that it is normal, but they want to know why. I think that clear communication from doctors would really help their confidence. (Doula 8) |
| Banke-Thomas et al. (2017) | Recognition of the importance of prenatal care | Prenatal care is important for the safety for the baby and the mom. If the baby has some issue, it is possible to know about it earlier with prenatal care. 22-year-old Burmese-speaking woman, Gravida 2, Para 1, first visit. |
|  | Positive drivers for use of prenatal care | My neighbor and I talked about her positive experience at the clinic and I felt encouraged by her advice. I made the decision on my own. 21-year-old Kinyarwanda-speaking woman from DR Congo. Gravida 1, Para 0, made 4 visits so far. |
|  | Barriers to prenatal care use | Language barrier was a problem for me with other clinics, and I felt very frustrated. I could not tell the doctor how I really felt, sometimes my husband had to go with me because he speaks better English, but I really did not like that and he sometimes did not have time. But not with this clinic, since there are so many interpreters here. 25-year-old Burmese-speaking woman,  Gravida 2, Para 1, made 3 visits so far. |
|  | Opinions of specialised prenatal care | I have been coming to the clinic for a long time, I have only gained profit from the clinic. I love everything, the taxi, the interpreter and the doctor. I love the interpreter because she  helps me understand the language that I don’t speak. Then she talks to the doctor that gives me the medicines that I need and cares for me. 22-year-old Somali-speaking woman, Gravida 1,  Para 0, made 4 visits so far, came to the USA in 2014. |
| Cameron et al. (2021) | Impacts of COVID-19 postnatal healthcare | “The hospital rules are strict during COVID. Visits are forbidden, friends can’t come, and they could not be there to help me. My husband was allowed to visit me twice a day in the hospital. I stayed for two days. I felt really lonely, it was a hard experience.” (Participant 3) |
|  | Loss of informal support | “It was really hard during COVID. In Syria I had my family… but to give birth here with no one with me?! It was really hard. I needed someone with me, my neighbours, my friends… I felt like I was drowning.” (Participant 7) |
|  | Anxiety and grief caused by COVID-19 | “I was scared of COVID. I was scared over my children’s health and because I had recently delivered, I was afraid of my last baby’s health.” (Participant 1). |
| Cameron et al. (2022) | The importance of social support | “They were cooking for us, they brought us food. One of my neighbours was helping me clean the home. They helped me with my baby boy as well. They would bathe him, dress him, they were really supportive and helpful. ”(Participant 4). |
|  | Impact of structural barriers on access to and quality of care | “I desperately tried to tell them that I wanted an epidural. I tried to explain to the doctor and doula by pointing to my back, but they could not understand. They thought I wanted them to massage my back. I suffered a lot. I was in labour for eight hours…I just cried. ”(Participant 7) |
|  | Provider paternalism and women’s decision-making autonomy | “In a meeting with my family doctor, she said that I was fine and there was no need for me to see a [mental health professional]. She said I knew how to care for my baby and there is no need to see someone. [Doctor] cancelled my appointment…I was very upset and disappointed because she didn’t know what I went through and how I felt. She wronged me, I needed her to listen to me…I really needed to see a mental health professional so they could help me. ” (Participant 9) |
|  | Valued and missing services | “I met the doula one month before I delivered... She didn’t leave me when I delivered my daughter. She stayed with me in the hospital. I was happy to have the doula because for me, I do have many friends, but I had no one to accompany me to the hospital.”(Participant 5). |
| Chrzan-Dętkoś & Murawska (2023) | The burden of traumatic stress on mothers and midwives | ID 2. I feel more burdened, even though I do not have much contact with people who have fled the war. That is because it also affects me – this war and what I see in my work. |
|  | Cultural differences | ID 5. There are not big cultural differences between our countries. Our religions are quite similar. They understand the cultural difference. We try to understand their culture and their religion. However, it does not matter to them. They want to feel safe |
|  | Challenges in working with female patients from Ukraine | ID 4. It is more challenging because of the language barrier. I know a little bit of the Russian language, so this is the way I try to communicate. I also have a colleague from Ukraine, and after she and the patient give consent, I call her, and she relays to the patient what I have to say, and vice versa. |
|  | The need for additional support in their workplace | ID 2. What we would need most are regulations and the financial issue – we’re trying to help, and we’re able to do it, but we need some support. It’s just not regulated by any laws. |
| Coe et al. (2024) | Building foundations for belonging | The things that we had to do during our pregnancy, like, going to present our belly or getting an ultrasound – all these things are very western culture … the natural remedies from our parents and grandparents, it’s helpful for me … they are important things for me. |
|  | Cultivating reciprocal curiosity | We have a lot of worries and concerns [during pregnancy]. But if we put our trust in [health professionals and interpreters] then we feel we are warm and have warmth. And we feel like we have protection |
|  | Storytelling as an expression of self & shared power | Sometimes in English, [health professionals and interpreters] would want a direct answer: yes or no. Some would understand [when we answer using stories], but some don’t. If they’re not culturally aware or sensitive, and they don’t really understand our communication style, it becomes a challenge. They get upset at you if you talk about something else … When they tell you off, you don’t really want to use them the next time. You don’t trust them. |
| Denzongpa & Nichols (2023) | Familial influences on maternal experiences | Leena (Case 1, age 37) describes: “My mother-in-law wrote my name in the hospital to get the operation (sterilization) done. I didn’t know any better at that time. … I should have ran away.” |
|  | Experiences of maternity-related care | The doctors here (US) are very polite when they talk, unlike over there (camp). They yell, get angry (doctors at the camp). A very few … umm … 5% over a 100 were ok. But 95% of them did not speak to us nicely at all. But here, they are extremely nice. When I was in labor and cried, the doctors there (at the camp) would get angry and yell at me. But here… they say, “You are such a good patient, you can do it.” They motivated me so much… I felt like giving birth to another baby (giggles). (Rita, Case III, age 29) |
| Dube et al. (2024) | Accessibility of care | One of the key things about the service is that you have a midwife on call 24/7 and you can pick up the phone and ask anything (Som-FG). |
|  | Women feeling accepted | They have also picked up a couple of words in our language. They have been listening. It’s good cultural understanding you know. They ask you what you want to wear during labour, and I think we get validated without discrimination of our culture. (Som-FG). |
|  | Value of relationality | Here you have one midwife and you have built rapport and trust. The doctor and the midwives from [the hospital], which is just anybody no relationship, no nothing, she doesn’t really know what I want, she doesn’t know what I need… there is no relationship whatsoever (Som-FG). |
|  | Service expansion & promotion | We need the service to be expanded. (Som-FG). |
| Dube et al. (2025) | Accessible and responsive care | Adapting that care to suit individuals… If mums are struggling to attend the clinic… it might be that you need to do a home visit… so that’s really around working with women’s needs (Suzie). |
|  | Understanding and valuing women’s needs | When women have RMGP care [they] are receiving care from midwives who are aware and prepared to support them in their unique way, whereas, when they’re going through the hospital system, midwives don’t understand (Jane). |
|  | Strong partnerships | I became the midwife for all of her family. The daughters, the daughters-in-law, and their midwife for their next births. It was lovely (Jas). |
| Due et al. (2022) | Continuity of care and relationships with healthcare providers | Each time you have to restart again. And you have to tell the same story, you have to... and yeah that kind of creates a kind of, yeah you don’t want to tell everything, because you need to, to get the relationship with your professional, and they don’t get it because you don’t see the same person each time. Each time you go there it’s someone different. (Naomi, refugee healthcare specialist) |
|  | Culturally and refugee responsive care | She [a midwife] asked me some question. She did ask me about my, how some women have a women circumcision in Africa. If I’m part of it, and ‘how did I feel?’. I think those question was personal, and I told her that, “I don’t feel comfortable of you asking me to answer those questions” … The other question that she asked me, she knows that African, they have all these domestic violence things. “Have I gone through anything that will cause damage to my having the baby?” I told her that, “I don’t need to talk to you about those things. I think I’m here for my health” …There, she just says that, “look, it’s part of the government need to know about you”, but Australia is a multicultural country. Everybody have their own culture. Whatever have happening with me, my own culture issue … It’s my privacy. (Refugee woman from Sierra Leone) |
|  | Women as equal decision‑makers in their perinatal care: The importance of consent and control | I wasn’t feel like I was listened to … it’s just like when you go, they tell you, “hop on the bed, check that, and come down. Oh, the baby is fine”. That’s it. Sign you off, and you leave … So I feel neglected, actually. |
|  | Perinatal healthcare experiences have long‑lasting wellbeing implications | [My perinatal care experience] was very hurtful… I think, with this experience I have, if I get pregnant again, I’ll not close my mouth again, because I went through a lot. |
| Erga-Johansen & Bondas (2023) | Feeling alone and scared – safeguarded by the multicultural doula | ‘Throughout my pregnancy I thought about childbirth. How would this turn out here in Norway? Because everything was new and different from our home country. Culture, language, everything. New system for me. I was scared, so scared’ (Participant 2). |
|  | Needing to be looked after – cared for by the multicultural doula | ‘…the doula was present, and it seemed like mum was present. She took care of me, showed love, and we need love. And it makes it easier to cope with the pain when you feel safe’ (Participant 3). |
|  | Not understanding the language – understanding with the multicultural doula | ‘I hope that there will be an offer for all women who do not have anyone here; to get a doula from the same culture. Language is very, very important; *if we speak the same language, it is much easier to communicate and understand what we feel and manage to explain*  *our feelings in the same way and be understood’ (Participant 4).* |
|  | Giving birth in a new and unfamiliar culture – the multicultural doula as a guide with the midwife | ‘I do not know the language. I was also a bit worried if I am in pain, how will I convey that I am in pain? Will they realise and understand  that I am in pain?’ (Participant 4). |
| Evans et al. (2022) | Feeling safe | ‘She says she wanted her own mother to be there to hold her hand, but that was not possible, so during labour she put out her hand and her midwife held it during the birth. This was hugely important to her.’ (V2) |
|  | Being treated fairly and equally | ‘The way they treat you, you are not fully a human being and not fully a woman.’ (W4)  ‘I came here legally. But people judge you, they say you had a child in order to stay. It hurts, and it’s not true...My culture also judges you...I was shunned by my Indian husband’s family because I talked about women’s rights. But here in the UK you have no rights...You just feel empty…I have to stand up for myself and do things for myself, society will let you down.’ (W4) |
|  | Building a future | ‘I want a community group to share stories and experiences of giving birth and being a woman.’ (W9) |
| Glavin & Sæteren (2016) | Inadequate integration into Norwegian society | “They didn’t understand anything when midwives told them that we should do this and that. They understood nothing. I think it is important to learn the Norwegian language before you get married! Yes, I recommend those who give birth or women who are from another country that they must learn the language.” |
|  | Need for and fear of a caesarean delivery | “No, because they do not survive, the women who give birth by caesarean delivery in Somalia. You are not okay when having surgery. I’ve heard from a lady who had given birth here in Norway that the doctors are good because they’ve studied. It’s not like that in Somalia, the doctors there are bad. If I have a caesarean delivery in Somalia, I do not know if it will work well.” |
|  | Family support around the postpartum period | “It was a little… quite lonely… sometimes when my husband was at work… then I think more… but the situation became better with time.” |
|  | Support from health services | *“At the maternity ward, they said I had to get up and take care of the baby myself and find my food and freshen up, but I just wanted to lie in my bed. I had done a big job, I had given birth to a baby and I needed rest.”* |
| Fair et al. (2021) | Appropriate and applicable | "I am going to use this to try to improve how I deliver care and to put the ladies [migrants] in a position of power" (UK5) |
|  | Made a difference | “To learn about their culture, the importance of their habits ... to know and understand them and to see them with empathy” (Greece2) |
|  | Training gaps | "Access to these trainings and good quality protocols would improve care" (Netherlands1) |
|  | Supportive care | “That was a start; I just wish it would continue to be a program just like that … As ORAMMA we had a very supportive role between structures and secondary  healthcare. We were a connecting link in facilitating their contact with secondary healthcare, where the camps’ system was lagging behind” (Greece1) |
|  | Working alongside peer supporters | "The supporter attended appointments at the hospital, community midwife clinics and at the family's home. She offered flexibility and some consistency, in difficult circumstances. This was so valuable for this family’s experience of maternity care” (UK3) |
|  | Challenges faced | “Unless they have a problem, which these women usually do not have, either with breastfeeding or generally with their postpartum period, they will not seek out a health professional, they do not have it in their routine, as we do. If they don't feel something bad, they won't do it.” (Greece4) |
| Gateri (2024) | Discrimination | I have noticed that when we refugee women visit the hospital when we are not well, or our babies [are not well], the health care providers dismiss our concerns as if they are not expected. It is as if they wait for our conditions to get worse for treatment to be provided. (R1, a refugee claimant)  Some refugee claimants had been denied care or neglected because they did not have OHIP. Due to the lack of proper communication between the hospital administrative staff (specifically the uninsured patients’ liaison) and CHCs, CHCs provide letters to all uninsured and refugee claimant patients to facilitate access to services outside the centre. (SP6, a social worker in a CHC) |
| Haith-Cooper & Bradshaw (2013) | Medical discourse | P8fg1 “And obviously, your normal role of the midwife, you do all your checks, making sure everything's fine. Mum's, baby's fine. Making sure, you know, that you're not letting asylum seeker take over your perspective.” |
|  | Managerial discourse | P10fg1. “are there any maternity guidelines in relation to asylum seekers” |
|  | Midwifery discourse | P12fg1 (social support) “it's not a midwife to sort it out, is it” |
| Hearn et al. (2023) | Knowledge sharing | “…we had the knowledge and the information that we needed to provide information for whatever the women came up with.”(201) |
|  | Bicultural family mentors – the critical link | “I think [the] bicultural [family mentor] is the absolute essence of the program.”(105) |
|  | Finding our own ways of working together | “For a team to work well, it really requires that everybody is on the same page, communicating well, and respecting each other and stuff like that. I think that has been the beauty of this program, and that is what has gelled it together, even during the pandemic period.”(101) |
|  | Power dynamics at the intersection of community and clinical knowledge | “…there were lots of discussions over scope of practice and whose job it is to inform certain things…”(303) |
|  | System capacity for change | “We’ve been talking about different ways of funding, you know, Victorian [a state in Australia] health services for years. It’s not flexible. It’s just not flexible…”(109) |
| Hearn et al. (2024) | Structural inequities and the toll of the pandemic | “. . .someone who is an asylum seeker with a history of trauma, who has had the support of perinatal mental health through the pregnancy [. . .] the recommendation is for an  extended stay to provide that care and evaluation, and to see the necessary supports, for them to transition from hospital to home. But of course, that person can’t stay while her  [other children] can’t come to the hospital [due to COVID-19 restrictions]. So of course,  while the [healthcare] recommendations are in place, it’s just not feasible when these  COVID-19 restrictions are in place with no flexibility.” (Professional, hospital-based) |
|  | Supportive infrastructure | “. . .it [government-funded pandemic payments] was enough. . . just enough. . . for food. . . for family. . . sometimes.” (Postnatal woman, Karen) |
|  | Cultural safety during the pandemic | “. . .the case manager from [community-based refugee health and social care organisation] called us and informed us with that [pandemic information]. They are calling us every time there is new information.” (Pregnant woman, Assyrian) |
| Henry et al. (2020) | Conceptions of pregnancy and childbirth, premigration experiences | “If I don’t notice anything, I don’t need to go to the doctor.” |
|  | The impact of premigration experiences on women’s perceptions of health care needs | That worried me most, having to go to a strange person, well, someone doing the birth whom I had never seen before. . . I had assumed that the same female doctor would look after me who had cared for me during the whole time. |
|  | Experiences of antenatal and obstetric care and compensation mechanism for access barriers | They tried to explain the treatment. They tried to explain, we are going to do this and that. But when she didn`t understand, they performed the procedure anyways. Without telling exactly, what they were going to do! |
| Higginbottom et al. (2013) | Personal agency | P11: And we have something also in our tribe. If you’re going to have the baby you don’t have to cry and do this all kind of this funny stuff, because they’re going to sing a song about you that you’re a chicken or something…You can’t cry. You can’t do this funny face and this kind of stuff. You have to be strong for it. |
|  | Resistance to health practices | P11: And we have something also in our tribe. If you’re going to have the baby you don’t have to cry and do this all kind of this funny stuff, because they’re going to sing a song about you that you’re a chicken or something…You can’t cry. You can’t do this funny face and this kind of stuff. You have to be strong for it. |
| Khaw et al. (2023) | Continuous individualised support | *I find the women that I support … it’s really just about not being alone and having continuity of care is the biggest thing I think for them…. we’re always there and they have access to us, pretty much 24 hours a day. Doula 5* |
|  | Social connectedness | *… in pregnancy it was a lot of effort to really communicate that I was there for her to support her… being with a woman through her labour and birth … completely creates that sense of trust and openness…I guess it really communicates to the woman that I’m really just there for her. Doula 6* |
|  | Creating safe spaces | *I think having that strong relationship in pregnancy is really what makes the labour and birth so special in the way that you don’t necessarily have to do anything in particular or holding space and having that person there to share that glance with or share that look with you to be like, “Everything’s okay. You’re in control," works wonders. Doula 1* |
|  | Cultural facilitator | … even though I wasn’t from her cultural background… I was still trying to really understand and ask those questions… “Why is that not accepted in your culture?”… she really appreciated being able to talk about all of that in a respectful space. *Doula 10* |
|  | Non-judgemental support | *I step back because it’s her relationship with health providers. But my view is understanding 100% behind that woman supporting what she wants, so even if she’s making decisions that I wouldn’t make … the whole point of the exercise is to support her power, her decision-making, support her choice. Doula 4* |
|  | Enhanced communication & rapport with providers | *…if I can’t speak their language, they can’t speak mine. I feel a little bit panicked…whereas I feel the doula was that bridge … if the doula was calm and comfortable and not threatened by me, I felt the woman wasn’t as threatened or scared… there was a few things I could ask the doula that she (migrant woman) didn’t have to answer - she could just focus on her labour. Midwife 10* |
|  | Making connections | *I’m a bit of glue … because our clients are so complex they’ll have … all sorts of other workers involved… a lot of those services don’t communicate with each other very well… because I’m the one who’s only with the client and my only job is the client…I’m the one who can kind of connect the dots for everybody else who’s not communicating properly. Doula 10* |
| Kirkdendall & Dutt (2023) | Isolation and alienated knowledge | I had good experiences with all six of my kids in the Thailand refugee camp. My seventh baby was born in [Georgia, US] a few months ago. It was up to the doctor what they would do because this was my first baby that was born in America, and I didn’t know anything. [Whatever] the doctor or the nurse told me to do during pregnancy and labor I did it all. All six of my children that were born in Thailand, my water broke naturally. [In the US] I accepted all the procedures that the doctor would do to me. (K’Paw) |
|  | Gendered disparities and structural inequities | I would like to learn and speak English a little bit so that it can make things easier for me. The second thing is I want to learn how to drive in the future. I have my permit to drive but I will wait until my baby is a little bit older for me to drive and to go back to school. Also, I would like to work when all my kids are grown up […] It is hard for me as a mother, I don’t know how to drive and speak English. I can’t go to my kids’ school and ask questions when I need to, and I can’t communicate with the doctor when I take my kids to their doctor’s appointments.  It’s hard for me. (K’Paw) |
|  | Community support and precarity | “They [Embrace] care about someone during pregnancy and after delivery, all is gone. That’s the challenge I find here.” (Kwau) |
| LaMancuso et al. (2016) | Agreeability and gratitude from “easy-going patients” | ‘‘No matter how many ways you ask, ‘Do you have any questions?,’ they always say, ‘No, I’m good.’ But are [they] really? Or, are they afraid to question almost because they are afraid of how I’m going to respond?’’ |
|  | ‘‘Can I Ask That?’’: Another View of Karen Perinatal Preference and Understanding | ‘‘Most of them, they don’t know their right. They don’t know their options, so that is why they don’t say anything until the people are gone and they tell you, Why [are] they doing that?’’ (Doula)  Most Karen or Burmese women grew up like that.  We are afraid of something all the time. So even though they are not here, their minds start. They grew up with the fear. So when they go to the hospital, they want to ask something, but they are afraid. They keep it in their heart because of the fear. |
|  | Karen Doulas: Patient Advocates on the Health Care Team | ‘‘I teach them…Next time, you ask in front of [the] doctor. I will translate it for you. Otherwise you will not get the information.’ (Doula)  ‘‘Having people who can translate the culture…, the doulas and translators as part of our practice help[s] us. They give us tools.’’ (medical provider) |
| Lazar et al. (2013) | Challenges in patient-provider communication | I think the Somali interpreters are, I don’t mean to sound pejorative, not as reliable as some of the other interpreters, because they’re filtering through the eyes of the tribe and what they need to do is to tell the patient what I tell them and I know that they don’t, they modify it, because I’ve had others chime in and say “that’s not what he said” so I’m suspicious that it’s one of the problems we have in communicating, with the interpreter putting her own two cents in. (Male OB/GYN, 18 yrs. Experience with Somalis/FGM) |
|  | Frustration with perceived Somali women’s resistance to obstetric interventions | Our sense of it is that they perceive we want to do C-sections because it is somehow faster, easier, or more financially rewarding for us, and that we don’t want to wait for the vaginal delivery, we just want to push them to have a C-section. We sense that they think we are very quick to jump on C-sections, perhaps because of the language barrier or the cultural barrier. I don’t feel that it is accurate. (Female OB/GYN, 6 yrs. experience) |
|  | Providers’ perception of mistrust by their Somali patients | I think they come in with some preconceived notions as well; that we’re forcing health care on them, we’re forcing tests on them that are unnecessary . . . so getting over that boundary, that’s a barrier. (Female nurse practitioner, 5 yrs. experience) |
|  | Suboptimal provider training in the care and management of women with FGC | You sort of get dropped into it, I think we try to talk when there’s a patient that we know is Somali whose having her 1st baby and is going to have a significant tear, I think we try to talk about how to manage that when we can. (Female OB/GYNB, resident 4 yrs. experience) |
| Leoniuk et al. (2025) | Language barriers | The most difficult thing is the language barrier. Although it seems that these languages are similar, it is difficult to communicate, especially with those patients who have only been there for a short time and have had no contact with the Polish language at all. (Midwife 5, 28 years of work experience) |
|  | Cultural barriers | I think most of them base their experience on their parents, mothers or grandmothers… they are more focused on natural childbirth, breastfeeding, everything more physiological. (Midwife 1, 11 months of work experience) |
|  | Educational barriers | Ladies don’t know how our system works, that when nothing happens, you go to the clinic, so I kind of understand these visits to the ER. (Midwife 12, 6 years and 6 months of work experience) |
|  | Psychological barriers | There was one woman who ended up in the maternity ward after 12 hours on the train, who had escaped from Ukraine and came to us straight to the maternity ward because she had simply started bleeding on the train. (Midwife 2, 11 months of work experience) |
| Lephard & Haith-Cooper (2016) | Pre-booking challenges | One woman had been in the UK for 3 years and had never seen a GP until she tried to register when pregnant. She experienced difficulties finding a GP that would accept her. |
|  | Inappropriate accommodation | ‘They give me a room (hotel room)…(It was) very small, it was smelling of cigarettes. The duvet was very dirty. The bed… the walls… everything was very dirty. (P3) |
|  | Being pregnant and dispersed | ‘I have to start again from zero... I was pregnant. And I was sicking (vomiting) all the time. They bring me here….I didn't have nobody here.’ (P5) |
|  | Being alone and pregnant | ‘They want to give me epidural and take me to theatre. They could do anything. I was by myself, didn’t have anybody. So I just accept what they want to do.’ (P3) |
|  | Not being asked or listened to | ‘I asked them we cancel the meeting until we get an interpreter… I didn’t understand you and you didn’t understand me’. She said, ‘No, it’s ok we can go on – you understand English’ (P3) |
| Lillrank (2015) | Good experiences and expressed appreciation toward the maternity care system | A: How did you experience the cooperation with the professionals?  Marina: Yes, the cooperation functioned very well. They explained everything very calmly and precisely and involved me in the entire process…  A: If we dwell on your visits to the maternity care clinic during you pregnancy, how were you explained about the pregnancy and the movements of the foetus and about the birth giving?  M: I was told everything in a very detailed way, I was told what to do and what to expect, I learned a lot, they also told me things [about my body] that I did not know previously.  A: You experienced that you also gained new knowledge and enough knowledge about your pregnancy and birth giving?  M: Yes, I am overall happy with this Finnish maternity care services and also about the birth giving because I compared it all the time to my two previous deliveries [in my native country]  where the circumstances were horrible. I was really very afraid of this third birth giving because I though it is going to be similar to my two earlier ones. But it was not. The staff was always close by and supported me during different phases of my pregnancy. I would say that I am really in ecstasy of this [birth giving] since I received so much help. |
|  | Dramatic experiences and disappointments with maternity care | ‘We arrived to the ER around eleven o’clock in the morning. But nobody took care of Saynab. So four o’clock in the afternoon, even when my wife was very sick and we had our little child with us… It was an upsetting and a very difficult situation… When nothing happened even when we were waiting … so then we just left and went home…  A: Did nobody take care?  Ahmed: No, [the nurse] said that all others wait too, you just have to wait. I said that this is a completely different situation, my wife is bleeding, she is ill… Nobody did react in that situation so we came home. Next day my wife was very sick and we were at home. Then on Sunday she said that everything has come out, so I realised that everything has gone [a miscarriage had happened]’. |
|  | Tragic experiences as a result of failures of the maternity care system | ‘[the hospital staff] underestimated me because I am a professional myself. I would have demanded better treatment if I would have been a different kind of Finn. But [they] did not recognise me as a Finn because of my [foreign] name. They underestimated several things but I know how things should have proceeded. This is my greatest disappointment’. |
| Lukasse et al. (2025) | Healthcare in country of origin | ‘If you give birth in a public hospital, you are treated like you are in a factory. I mean, there are many women giving birth there. The staff are angry and tired. Everybody understands that. But you are treated like you are a burden.’ (Participant 5) |
|  | High quality care in the new country | ‘For me it was like my mother or grandmother stood by my side. Like that. And it was easy to feel good. It was like she is not going to leave me in any case. She will make things good any way. It was so good.’ (Participant 6) |
|  | Challenges as a refugee | ‘We did not have a car… I had to give birth. Thankfully, the ambulance came. We tried to think of other alternatives. We had no other option. We did not know so much. We did not speak the language.’ (Participant 4) |
| Mendel et al. (2021) | Community support – Role of doulas | For me, they’re not only my clients. After all they become like my friends, my sister. Because I have one client . . . and she is a single mother without—no family support here. Her parents are not here. I was there for her . . . I told her then, because of everything that she went through, I would be there for her. But there’s a time when I would have to break down and cry with her. And I would do that (Doula 4). |
|  | Cross cultural comparisons | In Thailand she had her mother already beside her. She didn’t even have to care for her baby after delivery—her mother took care of everything. So when she came here, she was alone, and she had to walk. She is the one who has to figure out about the baby, and she is the one who has to take care of herself (Doula 6). |
|  | Experiences with maternity care system (priority needs) | loneliness is the other challenge they face because they don’t have anyone here. Like maybe husband, some families do have families, but most of them—the majority—they don’t have families here so they feel loneliness in pregnancy. They think of their moms, sisters, and other relatives and they think, ‘Oh, what will happen to me? What will happen to my baby here?’ (Doula 10). |
| Missal et al. (2016) | Limitations of support due to separation from family | *When you have a baby and go*  *home and [are] not used to stay[ing] at home, you get overwhelmed. Need to get out and talk to neighbors* |
|  | The importance of cultural and religious practices | *“Praying in the ear is a must. Dad is the preferred man to do it. The same prayer in both ears.”* |
|  | The desired relationships with nurses | *One nurse was the best. She was coming to visit me often. She taught me about the baby and to care for the baby. She was nice. She was talkative.”* |
|  | The fear of caesarean section | *“Many mothers say: ‘I am not the first-time mom, I do not see the clinical condition [reason] to do it.’* |
|  | Views on postpartum blues/depression | *One participant recalled how her husband*  *responded to her when she was crying: “What are you doing? My mom had 12 kids, never cried. You’re weak. She had 12 kids, and you had one or two, and you’re crying* |
| Nenko et al. (2024) | Challenges with caring for migrant women | In our hospital the main problem was that almost nobody knew the language and we were short-staffed. We were preparing all the patients from the gynaecology department for the surgical procedures, we had to take them to the OR [operating room], we had to bring them back and to take care of them. So, if we had many procedures and there was even one Ukrainian patient it was very difficult. […] If the patient did not speak Polish or did not understand us, we even did not have the time to use the online translator. It was very difficult. (F1, P4) |
|  | Making it work: midwives’ strategies in facilitating better care | So, I was doing this postnatal education classes and I was so frustrated. I would spend an hour and say almost nothing, because I was trying to translate. And I really wanted to explain these things, so they would gain something after my visit but…it was difficult. So, my husband has done this very nice dictionary with Polish, English and Ukrainian. […] I even had it framed after some time. He really wanted to contribute. (F5, P4) |
| Njenga (2022) | Communication and resource provision | “I had a good friendly doctor. She appeals more to my community; she was using words from my language so there was that connection.” |
|  | Participatory decision-making | “Africa doctors are good because they listen to you unlike here; they make all the decisions.” |
|  | Provider attitudes toward cultural practices | I felt like that doctor that was delivering was judging me. She even at one point even got another doctor to come and look as if it was something, it was like a circus that everybody could come and see. That’s how I felt even if she didn’t say that to me, I felt it!. |
|  | Understanding the US health care system | Why are they giving us a lot of medication? Why do I need all this medication? The doctor is supposed to treat me and make sure I don’t ever come back here again. Back home if we are sick, we go to the doctor, get few medicine, there you go! You are cured! |
|  | Mistrust of Western health care | “I did not like it (pelvic exams). I feel it is not good to keep checking down there so I stopped going for appointments. |
|  | Religious beliefs | “They told me I should have a cesarean section, but I say, ‘No, it’s Allah’s will, whatever Allah wills. He decide how baby come out.”’ |
| Njenga (2023) | It’s what women believe and do | “Being pregnant is normal. Getting pregnant and having kids is what women have to do.” |
| Olcoń et al. (2023) | Understanding the needs, experiences and identities of refugee and migrant women | “There’s a lot of racism in health sectors. I’ve seen it quite often working as a midwife” |
|  | Improving access and quality of services | “No one ever knows who we are or what we’re doing” |
|  | Taking an individualised approach | “It’s important for us to remind ourselves that we are not here to save them. They don’t need saving. We’re here to support them and be that guiding hand when they need it. Because they have survived all this time without us. They have the tools themselves.” |
|  | Using interpreters | No quote provided in study |
| Owens, Dandy & Hancock (2016) | Social support | ‘‘I don’t have a very close friend, just if I go to a service like here, just meet with people, like interpreter, those things’’ (woman 8 via bicultural worker). |
|  | Gaining of knowledge | ‘‘you know I don’t have experience, the first, and they give me a lot . . . like every single thing,. . . they give me like that, that knowledge’’ (woman 9). |
|  | A holistic service | ‘‘I’m very happy with the process, and even after giving birth, there is a continuation’ and ‘‘the service is complete’’ (woman 1, via interpreter). |
|  | New opportunities | in my country father can’t come to delivery room. But I don’t know why, I think it’s very good, I like father being there, and my husband come to delivery room, it’s good for me, very good,. ...he’s always talk about delivery room, and he has good memories about see baby coming (laughs) (woman 4). |
| Papadakaki et al. (2021) | Low capacity to meet the health care needs of migrants in a culturally appropriate manner | “... No one is allowed to be with the woman in the room during labor. Women are very fragile at that moment and they need their own people. They don’t even understand the doctors’ language . ” (Interpretation Service, FG2) |
|  | Doctor-centered system with minimal investment in the health care team | “Midwives are very emotional and the main problem is that we cannot help these women with the social determinants. It would be very helpful to have social services for helping them find a job, for helping their families and especially their children” (Midwife, FG1) |
|  | Lack of service integration and continuity of care | “The link between primary care facilities and hospitals is still lacking... we face difficulties in formal procedures” (PHC society, FG2) |
|  | Low engagement during crisis—service providers’ burn out | “It is worthy to offer assistance to a woman who suffered so much, but we are very few and unsupported. We need more support from the system. All those providers working with vulnerable populations like refugees or other traumatized individuals, spent a huge amount of effort to care for these people, help them feel safe and meet their needs. We sometimes disregard our own needs and get exhausted.” (NGO, FG2) |
| Pierce et al. (2025) | Pressures on HCPs and COs in delivering care | “I should be able to quote [the guidance], but I can’t. I’d have to go in and check it and that doesn’t feel great [HCP10].” |
|  | How HCPs and COs adapt care to navigate challenges | “sometimes I’ll see pregnant women off the record when it’s not strictly regarding maternity care, especially if there is any doubt about whether or not they are entitled to care. It worries me because I know it’s something that I could potentially lose my licence over, but my role has [been], and always will be, to provide care [HCP16].” |
| Riggs et al. (2017) | Learning together: informed, prepared, confident, and reassured | All of the information I have learnt, and all the new information I have, I feel stronger … so I feel like I know more about what’s happening |
|  | Social and emotional support: sharing stories and experiences | The best thing about coming was seeing each other, sharing stories, sharing problems. And hearing stories, hearing other people’s journeys, I liked hearing them very much. |
|  | Trusting relationships: continuity of care and care provider | When we were in our country or in the camp, after we delivered the baby, when we went home no one came and visited us. Here, after we went home, we have our nurses come and visit us after 2 days. They came and check on us, and after that she [the bicultural worker] visited us. |
|  | Challenges in the hospital: communication and privacy | Other doctors and nurses would come in and that was really uncomfortable for me. They didn’t do anything, but they looked, and that was really uncomfortable. |
| Rogers et al. (2021) | Supporting access to health and community-based services | We provide information during the pregnancy and into transition into parenting and also link them to playgroup, community support or and also to provide any culturally appropriate information or education related to pregnancy and parenting. …advocating for them on behalf of them. (CCW) |
|  | Improving the healthcare experience | I definitely find that my experience with the women who are labouring who have been part of the groups, they seem to be more educated about their birth and what to expect... I’m meeting them for the firs time at three o’clock in the morning, and people that haven’t been part of any antenatal education …struggle a lot to understand their state, what their body is going through. So, I think they (CCWs) help facilitate that kind of empowerment for the women. (Doctor1) |
|  | Organisational factors affecting CCW service provision | They (CCWs) only both work two days a week at the moment. I would say there’s enough work for four days a week each…So, it’s looking at how much work is there at the moment and how much more they could do if they had more time. (Midwife4) |
| Rowe et al. (2023) | Women’s understanding of health and wellbeing during pregnancy and after childbirth | *‘Yeah, for me you should have really good support. For the people like me, they do not know anything. There should have somebody to guide them, to tell them the things. Like the volunteers who work here, I have not seen in London. So, midwife or whatever, they should guide them properly in a proper way. So, it’s going to be such a beneficial for the healthy pregnancy’. (KII 6)* |
|  | Women’s specific health needs: cultural and language challenges | *When I had my first baby, it was very difficult, you know, very, very difficult. And I did not know how to say even a word, because I did not know no English at all. And because of pregnancy, because the labour was very difficult, and they were even saying to put me, you know, on C-section, but I was a… because I do not speak English, they did not know what to do, because I had to sign some consent like things like that. And it was very shocking. I was just crying, because when you do not understand anything. (FGD P3)* |
|  | The experiences of women seeking maternity care in terms of maternal multimorbidity | *‘When I was pregnant, I used to go when I had appointment. But nobody used to discuss with me about my mental health and anything. They just used to examine, the basic examinations for pregnant lady. They used to do the weight and I really do not remember what they do. But they do not talk about my mental health, they never discussed whichever problems I had.’ (KII 6)* |
|  | Support from health and social care provider systems and charitable organisations | *‘The social worker helped me. She called on behalf of me to the migrant help, to the Home Office and everything. She helped me really. And thanks God. We got the asylum support and they moved us to the Liverpool and Children centre, they helped us a lot. So, after having support from everywhere, I felt like very confident. Before, I felt that I am really alone. I do not have anybody, I do not have any support, I do not have anybody. But when they start supporting me, everybody was coming and visiting us-people from Children centre, the health visitor, the social worker they were looking after (baby X) and me and everything’. (KII 6)* |
| Russo et al. (2015) | Satisfaction with services | “…the health system here is really…very nice…[in Afghanistan] most of people just don’t go to doctor… they just fall pregnant and…give birth at home as well…here they do ultrasounds, blood tests, glucose tests, everything was done to take care of me and my child. But there, nothing…” (Zamira, one child born in Australia, and pregnant with second child. Living in Australia for 5 years) |
|  | Interaction with health staff | “..[when I was pregnant]…the nurse came to me I start crying. I don’t know why and she really listen to me, and she was just so good to me. And after I cried, my heart was like empty. I felt so good and I thought it is just really nice to have someone to understand those kind of situations. I love it.” (Saera, two children, both born in Australia. Living in Australia for 5 years) |
|  | Barriers to seeking support in formal settings | “I felt like I was judged by my doctor…I wanted to do things according to my tradition but I was expected to do things differently.” (Focus group participant) |
|  | Family and female kin | “It was hard to be alone here…because I see my sister and my brothers wives in [Afghanistan] and there was support from everywhere like family…they get lots of help and the difference only was that I didn’t have my family around me…” (Saera, mother of two) |
|  | Culture, traditions, and community | “Raising children by myself is very hard here. If I was back in Afghanistan, my kids would grow up with cousins and I would live with extended family…the work load and responsibilities shared…I am very scared that when my children group up they will have no memories of their culture…” (Feroza, mother of one and pregnant with second child) |
|  | Changing roles of men | “…[I] like Australia because the responsibility of raising children is shared [with husbands] and fathers are expected to do as much as mothers…” (Focus group participant) |
|  | Enhancing connection to improve emotional wellbeing | “I joined a group in my area and within a few months I felt like I was getting better…I realised that a lot of women in the group were having the same emotions like me and that I wasn’t alone.” (Feroza, mother of one and pregnant with second child) |
| Smith et al. (2025) | Interpersonal caring | *…things I like about having a baby here is that they take good care of you like your mom. Close, like your mom is close to you. — Nepali Participant* |
|  | Flaws in US maternity care are amplified for refugees | *They tell you, “Oh, we'll be back in a minute,” and they give you the sign where you can beep if you have some emergency, and you call them sometime you feel thirsty. Sometimes you have to go into the bathroom, and they never come back like somebody will come back in 5, 6 hours like when another shift started, and if you if you call them, they will receive the call, and they say, “Oh, we'll be there shortly.” And then they take half an hour. — Nepali Participant* |
|  | Multidimensionality effects knowledge, preferences, and expectations | *I told her “I don’t want the cold water,” so I asked her for the cold water to be hot water, she said, "Cold water?” “No, I want hot water.” So she said, “Okay,” and went to get the hot water. And also, the shower put out cold water as well. For us after delivery, we want hot water. So, our parents when we get home they warm the water, so we will shower with hot water, not cold water. — Karenni Participant* |
|  | Complexity of the US health system combined with unfamiliarity contributes to lack of confidence | *We go to see our baby doctor, but when I give my daughter the nipple she [doctor] says it’s not right, “when you  give your daughter the nipple you have to put the whole thing.” I told my husband “I can’t do that!” Because in our country ladies do not do it like that [lots of laughter]. It’s different. It’s not easy here. You have to hold the baby like that [gestures holding a baby to the side instead of upright]. I don’t know. It’s not right [more laughter]. I’m about to cry. I can’t do it like that. I’m so tired! —Karen* |
|  | Problems with language interpretation | *When he interpreted in-person, he wouldn’t let me ask questions to the doctor. When I asked or tried to ask questions, he became impatient and mean. — Karen Participant referencing dilemmas with a male interpreter* |
| Stapleton et al. (2013) | Service provision: models of care, access and appointments | Women do end up ringing me or dropping in. They know that I work in this particular room and come in [. . .] because they’ve lost a form [. . .] or they’re looking for a bit of support. [. . .] I don’t say no to those women, so that takes time. (Clinic Staff) |
|  | Socio-cultural and medical norms: differences between home and host country | In our culture, when you’re really in the pain [. . .] we prefer to pray and ask God for help and see Doctor there. Doctor and you and the God [. . .] we don’t need anyone else, that’s all (PRA 1). |
| Tankink et al. (2024) | Language and interpreters | “And I’m thinking about what you [the interviewer] just said, ‘don’t you find it difficult with an interpreter?’ But for us – I often talk to someone else, a sister or a friend or a child, so a [professional] interpreter can truly be a luxury, that what I am saying gets translated at least. Because with someone else – I say ten sentences and then they translate one thing, and then the lady says ‘yes’. Well, that was it (laughs). So I truly think the [professional] interpreter is a luxury!” (Midwife 6). |
|  | Cultural differences | “Usually, people from Syria are very motivated; they try to learn Dutch by themselves; they are well informed about everything, always on time. Well,  from Eritrea, not everyone is like that, of course. There are also people who arrive on time and show up consistently, so not everyone – but there is a difference…”  (Midwife 8). |
|  | Building trust | “And if someone truly does not want to share anything, then it is just done, then I always say, ‘I know you have been through something rough, I know it’s a  process that can be painful but also beautiful. Know that I’m here for you, the moment you do want to talk about it. I’m here for you.’ And then – often that is just enough to open up a story.” (Midwife 5). |
|  | Relocations of asylum seekers | And as a result [of relocations], you simply lose control from time to time. And that is where I struggle most, thinking ‘oh, so now you are gone and going somewhere else, is it going to be handled in the right way?’ (Midwife 1). |
|  | Delays in access to care | “I once experienced a situation where a woman had a lot of blood loss, but she only spoke – well, I don’t know which language, but let’s say Tigrinya, and  there was no one around her at that moment who could speak English. So it took about two hours until someone was able to call me on her behalf. By the  time I arrived, it was already too late because there was indeed something wrong with the baby, causing her excessive bleeding. But because she simply didn’t know how to reach us due to a language barrier, she just didn’t call. And things went wrong.” (Midwife 11). |
|  | Interdisciplinary collaboration | “Actually, it sounds very rough, but a part of the humane – is lacking there [in the hospital], and that truly breaks my heart, when someone just says afterwards:  ‘but that was no discussed with me at all, I didn’t want that, and I had put it in a plan with you.’ It just breaks me when I see that, and the communication as well – not much effort is made to guide them [the clients] in a proper and correct way. And that puts them – that is a very vulnerable position as a birthing woman.” (Midwife 5). |
|  | Housing conditions | “If you see someone saying that she feels depressed because of the burden from COA, that she may be deported, that she will be relocated to another center and that she feels unsafe, that she cannot return to her country of origin because she would be threatened  with death. When you witness that happening right in front of you, you think, ‘Oh my God, that woman truly cannot go back because she will simply be killed. (…) Yeah, it truly moves you’” (Midwife 3). |
|  | Resettlement after forced migration | “As soon as they [forcibly displaced women] leave the asylum center, they are suddenly assumed to be normal people. They are still the same individuals who don’t understand certain things, and there are still things that need to be arranged.” (Midwife 6). |
|  | Prevalent mental health issues | “Of course, that is not our profession. And it’s also too intense, you know, sometimes the tears are almost in your eyes when you hear what they have  been through.” (Midwife 2). |
|  | There is an imbalance between midwives’ responsibilities to care for the women and the resources available (shortage of resources) | “[When caring for forcibly displaced women] you feel like a social worker, you feel like a planner, you feel like someone’s buddy, you feel like a psychologist– you just have a lot more roles than only your profession.”  (Midwife 3). |
| Tobin & Murphy-Lawless (2014) | Barriers to communication | “It’s just, you try to do it visually, you know smile and reassure them, rub them, things like that, you know try to make them at ease, show them how to breathe, things like that … she had no English at all and she was on her own, very young, 17 year old girl … she didn’t know what was going on, you know and she was in pain, you know she did okay, we got through it and that but I just remember thinking oh this is just horrible”. |
|  | Understanding cultural difference | “But you might kind of say well why didn’t you go to a doctor, you kind of question them, you know what I mean, but no more than if it was an Irish woman, I’d say well do you not realize the importance of antenatal care, I would be the same, I would get a bit annoyed but I would be the same if there was an Irish woman, you know, because like you just don’t know, if they’re high risk and they are HIV-positive … and I suppose I’m being a little bit racist or whatever”. |
|  | Challenges of caring for women who were unbooked | “I think you get, that’s where you get a little bit of your racism sort of coming in then, and you know just not even racism, but just what are they doing here. And that’s why I feel if there was some sort of study day, just to fill people in, we’ve had asylum seekers in who have had horrific things done to them, you know in their own home countries and we can physically see that.” |
|  | The emotional cost of caring | “How does it affect me, you just feel sad you know, but you just do the best that you can and that’s all you can do”. |
|  | Structural barriers to effective care | “Women that were maybe high risk would be transferring to other parts of the country, so when their babies were born there was no neonatal services available to them and they’d have to be transferred back up into Dublin or Cork or wherever the main centers would be so it was absolutely crazy from every perspective”. |
| Tobin et al. (2014) | Lack of communication, connection and culturally competent care | Scene 1  When she arrived [by ambulance] she was feeling really bad. She understood nothing when she arrived at the hospital.  Act/Agency  The doctor didn't touch her. He just looked at her files. [He told her] to go back to the hostel. Because she came by ambulance she had no contact number for the hostel , she didn't know where she was going.  Agent  [Zita Crying] She went to reception but they could not help her…She was just so distressed she just didn't know what to  do…She went outside and lay on a bench near the hospital ,she asked people passing could they help, did they speak any French and no, nobody could help… she stayed there the whole day.  Co-Agent  She started to vomit and apparently a woman stopped. She said “Why are you getting sick? ‘I'm not well they told me to go back To the hostel’, but she explained she didn't know where (that  area) was. She had a paper with the hospital name so the woman took it from her. She was already showing signs of  blood on her clothes… [Zita Crying]…she said the woman said we'll have to go back there. So the woman said to her, ‘as you can't walk I'll get an ambulance’. She spoke French and took her back to the hospital. She started to explain everything to the staff, the lady. They admitted her straight to the labour ward,  the lady stayed with her until she gave birth. |
| Toke et al. (2024) | Care design and accessibility | *“So when I have my relatives in the appointment with me they can help me by asking questions and thinking of what to say or talk about. What we can’t think of, they can help think for us and ask for us.” —Participant 7* |
|  | Promoting choice and control | *“If a [pregnant woman] feels like their care provider is good, it’s someone who speaks nicely. I feel like when you get a care provider like that, your pain and problems are already half gone.” —Participant 4* |
|  | Trauma-informed interpreting | *“The health professional asked me whether I understood the interpreter and I said no, not really, he didn’t accurately interpret what you’re saying and what I’m saying. She said to me: because you couldn’t understand him, I will not call him again. There won’t be any difficult words for you to understand. Your husband will be able to understand also. . . I felt good.” —Participant 4* |
| Verschuuren et al. (2023) | Interdisciplinary collaboration | “Collaboration with the GZA and the COA [is the most important challenge in perinatal care for AS]. The last couple of years, the general opinion of GZA and COA has been: people are autonomous and should take care of their own business. Being involved [with the client] is labelled as ‘unprofessional’.” -Participant 33. |
|  | Communication with clients | “The costs of using telephone interpreter services [for RRP] are such, that we decided to not use these any longer. Most of the time people know someone who speaks their language and who also knows English or sometimes Dutch. Then we call through them. Or we use Google Translate.” –Participant  101. |
|  | Continuity of care | “Sometimes COA forgets to inform us when a pregnant woman is going to be relocated to another center or sent back to her country of origin. In that case we only find out when she does not turn up for her consultation. That cannot be right”. Participant 37 |
|  | Psychosocial care | No quote provided |
|  | Vulnerable situation | “I oftentimes feel like I fall short, especially on a social and emotional level.” –Participant 69. |
| Willey et al. (2018) | How to identify women from a refugee background | …I find it difficult to ask…you sometimes feel like you’re prying… I sort of probably should ask more. [FG6] |
|  | The MCH nurse role when working with families from a refugee background | P1 . . .sometimes it’s hard to tee up the visit because you don’t have an interpreter to tee up the visit . . . you try and ring and make the appointment and book the interpreter but whether the person actually knows that you’re going to come at that time and whether they’re going to be home. . .  P2 . . .sometimes it’s easier to hop in the car and say ‘I’m a nurse’, I find a calendar and the right time . . . and then say ‘interpreter’ and they go ‘yes’. I just find it so much easier. [FG5] |
|  | Interpreting issues | Q Do you have access to speaker phones or do you use your own phones?  P2 We don’t do it at all.  P4 It doesn’t work actually, does it? They don’t want to talk into the phone. They want to have face-to face.  P1 And you need something clearer. We’ve got little old phones that are not very good for those sort of things.  P3 That would only work in the centre and we’re often home visiting. So most interpreting goes through a  family member . . . [FG1] |
|  | Access to other referral agencies | . . .The neighbourhood House or . . . playgroup. . .I think this Baptist Church has a few programs for them as well. . .And the Salvation Army. . . they have a community garden that a lot of the men work at, . . . So there’s a few different programs for different areas of the community that are set up . . . [FG6] |
| Winn et al. (2018) | Pregnant refugees are a heterogeneous population facing multiple barriers to care | “P5: I know how distressing it is for them [refugees] to be in a new country, not understanding the language, not understanding the culture and the policy, having to come for so many medical and pregnancy related appointments, and sometimes they ask the question why? Why the blood works, why the testing, why the ultrasounds, why they make me come back every month, and then twice a month, and then every week in the last trimester?” |
|  | Health care professionals specialized in refugee health engage in diverse strategies of care | P5: “I always use highlighters, highlight the relevant information, and then print a navigation map, what bus you take, where do you get off. I circle everything, I’m always telling them if you are ever lost you just show this to the driver, they will know exactly where to tell you to get off. When I’m calling the agencies, I’m preparing them to make sure they know my patient doesn’t speak the language. [I ask] do you have anybody in the agency who speaks that particular language, any volunteer, anybody? If not, I’m asking [the refugee], please bring your interpreter with you if you can, and I’m always trying to tell them, bring an adult interpreter. We do not want for children to be exposed to that you know, taking on the families problems, because perhaps it becomes heavy for them.” |
|  | Funding cuts created a confusing system which jeopardized care | “P2: We heavily involved like, social work to figure it out [levels of coverage], like all of the front staff, and they had to be like on the ball. And then things kept on changing […] so it just made it very confusing […] Yeah, so it was just constant energy, umm involved in this.” |
|  | Syrian influx created additional strains on existing problems | “P2: It’s been super busy […] the Syrians doubled our numbers, so we’re completely maxed out. Lots of pregnant women within the Syrian Refugee community.” |
|  | Health care professionals unfamiliar with refugee health may be overwhelmed | “P9: Sometimes our [refugee] patients even ask us in triage like financial concerns, and I don’t know what to say at all. Like that’s something I would like to be more educated on, like what kind of services are available to you [refugees]. |
| Worabo et al. (2024) | Maternal healthcare experiences | *“Then the nurses and doctors, when they found out that my husband was not there, they were trying to comfort me. It was a really good experience. I didn’t even realize my husband was not there. I was really happy that they were so mindful and were there for me. They would make sure that I understand and explain the steps to me, like, ‘If things go wrong, then we will probably do a cesarean-section.’ They would tell me ahead of the time and make sure that I understand, and then explain the steps” (Participant 3).* |
|  | Communication | *“I took my son with me to the appointment, and I was having some trouble. When I was there, the interpreter translated my message completely differently to the doctor, and my son noticed and told him, ’Hey, that’s wrong. That’s not what my mom is saying.’ Then the interpreter got mad at the little kid, ‘How dare you tell me I’m wrong? I’m the interpreter. I know what I’m saying.’ Yeah, we had a big problem, not with the doctor or the staff, but the interpreter was not communicating the message right” (Participant 2).* |
|  | Access to care | *“The biggest challenge for women in the Afghan community is transportation. Most of us do not drive, so we cannot make the doctor’s appointments. We must wait for our husbands” (Participant 17).* |
| Yelland et al. (2014) | Language services in the context of care | My wife … wanted the interpreter to be a female but he was male. It was very shameful and uncomfortable for my wife. She told me she asked for a female interpreter but they didn’t listen to her. She couldn’t tell her personal problems to a male interpreter as in our culture it’s not right. She was very upset about that. (Male participant) |
|  | Women and men’s experiences of being asked about social health issues | Yes I can talk to them [health professionals in pregnancy] about my health problem but don’t feel  comfortable sharing with them my personal and domestic affairs … one thing I understand is that it is not part of their job to listen to our family problems.  (Female participant) |
|  | Identifying and responding to social health issues: Identification of refugee background | We ask them about what sort of accommodation they’re living in, what they and their partner’s employment status is, often then it comes out if they’re  not working or they’re on benefits or whatever the situation is, but we don’t specifically ask them if they’re refugees. (Midwife) |
| Yeo et al. (2024) | Perceptions toward hospital and prenatal care | “If they think that their health is good, they are not going (to the prenatal care).  (Interviewee 10, Afghanistan)” |
|  | Life skills | “I know how to take a bus and go somewhere that I know. If I know about the place before. Like, I know how to take a bus to that place, but for places that I don’t know or I haven’t been I can’t because I cannot read the map.” (Interviewee 9, Afghanistan) |
|  | Language proficiency | “I still don’t feel able or comfortable plus, you know, when you make an appointment for sickness or something, they ask why do we need to see your son today. You need to be specific and give the exact symptoms or so I don’t feel I am at the level to tell them.” (Interviewee 16, Syria) |
|  | Cultural norms and practices | “In our tradition, in our culture, we prefer women to take care of us.” (Interviewee 4, Syria) |
|  | Social support and network | “When you first come here you don’t know anything, so they (the refugee resettlement agency) helped us, showing us, or telling us about the insurance, how to go to the doctor, or they took us to the doctor, social security administration office, all this stuff.” (Interviewee 16, Syria) |
|  | The characteristics of health care providers | “They were really good. Even though I come from different background, I wear a scarf (Hijab) but that wasn’t like an issue. They were so respectful of my needs and understanding that as like a veiled woman. I had to go through surgery at a certain point. So if by accident, my hair is showing, they will cover my hair. So they didn’t let me feel that I’m a stranger.”  (Interviewee 13, Syria) |

## Appendix D: Table with findings, categories and synthesised findings

| **Findings (n=122)** | **Categories and accompanying descriptions (n=4)** | **Synthesised finding 1 and explanatory statement** |
| --- | --- | --- |
| Culturally and refugee responsive care (U) | Cultural differences in health practices  Traditional cultural and religious beliefs play important roles in shaping perinatal experiences for women of refugee backgrounds. These beliefs influence how pregnancy, childbirth and postpartum care are understood and approached. Many women view pregnancy and childbirth as natural life events that should involve minimal medical intervention. This worldview can contribute to discomfort with, mistrust of, or avoidance of Western healthcare systems, where pregnancy and birth in these countries are often medicalised and obstetric interventions such as caesarean delivery are normalised.  Tensions arise when Western models of care conflict with traditional cultural practices, leaving women feeling that their cultural beliefs are not acknowledged or respected by healthcare professionals. For many, childbirth and the postpartum period are deeply meaningful and sacred life transitions. Being separated from familiar cultural practices and support structures during this time can profoundly impact women’s experience of having a baby in a new country.  Women expressed a strong preference for being cared for by female health and social service professional staff, including interpreters and doctors, especially those who share their culture, language, or religion. In some cultures, families play a central role in shaping maternal care preferences, experiences, and family planning decisions. | Structural and systemic factors impact the provision of and access to equity-oriented maternity care  Healthcare professionals and women of refugee background face a range of structural and systemic enablers and barriers that influence the provision and access of culturally responsive and equitable maternity care. These factors are interrelated and span individual, interpersonal, organisational, and policy levels, shaping care experiences in complex ways. The main structural and systemic barrier shared between healthcare professionals and women is rooted in language and communication. |
| Provider attitudes toward cultural practices (C) |  |  |
| Socio-cultural and medical norms: differences between home and host country (U) |  |  |
| Culture, traditions, and community (U) |  |  |
| Cultural norms and practices (U) |  |  |
| Barriers to seeking support in formal settings (U) |  |  |
| It’s what women believe and do (U) |  |  |
| Religious beliefs (U) |  |  |
| Stigma and fear discourage seeking postpartum mental health care (U) |  |  |
| Lack of communication, connection and culturally competent care (U) |  |  |
| Interest in a language concordant postpartum navigator (U) |  |  |
| Familial influences on maternal experiences (U) |  |  |
| Outright refusal of care (U) |  |  |
| Maternal healthcare experiences (U) |  |  |
| Intentionally not seeking or misleading prenatal care (U) |  |  |
| Resistance to health practices (U) |  |  |
| Dramatic experiences and disappointments with maternity care (U) |  |  |
| Mistrust of Western health care (U) |  |  |
| Delayed hospital arrival during labour (U) |  |  |
| Barriers in interpretation undermine postpartum care recommendations (U) |  |  |
| Recognition of the importance of prenatal care (U) |  |  |
| Need for and fear of a caesarean delivery (U) |  |  |
| The importance of cultural and religious practices (U) |  |  |
| The fear of caesarean section (U) |  |  |
| Views on postpartum blues/depression (U) |  |  |
| Women’s specific health needs: cultural and language challenges (U) |  |  |
| Cultural differences (U) |  |  |
| The characteristics of health care providers (U) | Structural and systemic factors to equitable maternity care  Women were generally satisfied with the maternity care systems in countries of settlement and appreciated the medical and physical care they received. The healthcare systems were seen as high quality, and women liked the affordability of the public health care system and the facilities offered. Women felt they were treated with care and respect by health professionals. Specialised refugee health clinics and services were highly valued by women as they provided continuity of care and a holistic service. Women who did have negative experiences with maternity care systems reported language and communication barriers, particularly the lack of access to professional interpreters and resources in their native language. Having negative experiences with the maternity care system has the potential to affect women’s future access to services and has implications for the health and wellbeing of themselves and their child. |  |
| Good experiences and expressed appreciation toward the maternity care system (U) |  |  |
| Satisfaction with services (U) |  |  |
| A holistic service (U) |  |  |
| Agreeability and gratitude from “easy going patient” (U) |  |  |
| Valued and missing services (U) |  |  |
| High quality care in the new country (U) |  |  |
| Service provision: models of care, access and appointments (C) |  |  |
| Perinatal healthcare experiences have long-lasting wellbeing implications (U) |  |  |
| Lack of communication, connection and culturally competent care (U) |  |  |
| Dramatic experiences and disappointments with maternity care (U) |  |  |
| Pre-booking challenges (U) |  |  |
| Challenges as a refugee (U) |  |  |
| Experiences of structural inequities in maternal healthcare services (U) |  |  |
| Mistrust of Western health care (U) |  |  |
| Changing hospitals and/or providers when care has commenced (U) |  |  |
| Barriers in interpretation undermine postpartum care recommendations (U) |  |  |
| Lack of comprehensible postpartum information (U) |  |  |
| Language services in the context of care (U) |  |  |
| Experiences of antenatal and obstetric care and compensation mechanism for access barriers (U) |  |  |
| Tragic experiences as a result of failures of the maternity care system (C) |  |  |
| Impact of structural barriers on access to and quality of care (U) |  |  |
| Inappropriate accommodation (U) |  |  |
| Gendered disparities and structural inequities (U) |  |  |
| Understanding the US health care system (C) |  |  |
| Culturally and refugee responsive care (U) |  |  |
| Accessibility of care (U) |  |  |
| Service expansion & promotion (U) |  |  |
| Accessible and responsive care (U) |  |  |
| Barriers to prenatal care use (U) |  |  |
| Opinions of specialised prenatal care (U) |  |  |
| Inadequate integration into Norwegian society (U) |  |  |
| Communication (U) |  |  |
| Access to care (U) |  |  |
| Flaws in US maternity care are amplified for refugees (U) |  |  |
| Problems with language interpretation (U) |  |  |
| The experiences of women seeking maternity care in terms of maternal multimorbidity (U) |  |  |
| Care design and accessibility (U) |  |  |
| Trauma-informed interpreting (U) |  |  |
| Supportive infrastructure (U) |  |  |
| Prevalent mental health issues (U) | Capability building in the workforce  Healthcare professionals who do not work in refugee-specific maternity care settings often lack awareness and understanding of the unique needs and cultural beliefs and customs of women of refugee backgrounds, particularly in relation to pregnancy and childbirth. This limited cultural competency can lead to ignorance and can manifest as covert racism. Many health professionals are not adequately trained to provide refugee-specific care and are often hesitant to engage with women about their refugee backgrounds, leading to missed opportunities for appropriate, personalised support. There is a need for specialised training to equip healthcare professionals working in mainstream services with appropriate skills and knowledge to provide culturally responsive and tailored care. This will help prepare health professionals to better understand the complex experiences of women of refugee backgrounds. Furthermore, healthcare professionals may need to employ diverse and innovative communication strategies to ensure effective communication with their patients and clients, and that the health information provided is understood. |  |
| Health care professionals unfamiliar with refugee health may be overwhelmed (U) |  |  |
| How to identify women from a refugee background (U) |  |  |
| Identifying and responding to social health issues: identification of refugee background (U) |  |  |
| Understanding cultural difference (U) |  |  |
| Understanding the needs, experiences and identities of refugee and migrant women (NS) |  |  |
| Training gaps (U) |  |  |
| Health care professionals specialised in refugee health engage in diverse strategies of care (U) |  |  |
| Suboptimal provider training in the care and management of women with FGC (U) |  |  |
| Made a difference (C) |  |  |
| Appropriate and applicable (U) |  |  |
| The MCH nurse role when working with families from a refugee background (C) |  |  |
| Understanding and valuing women’s needs (U) |  |  |
| Suggestions to improve support for immigrant clients (U) |  |  |
| The need for additional support in their workplace (U) |  |  |
| Making it work: midwives’ strategies in facilitating better care (U) |  |  |
| Low capacity to meet the health care needs of migrants in a culturally appropriate manner (U) |  |  |
| How healthcare professionals and community organisations adapt care to navigate challenges (U) |  |  |
| Resettlement after forced migration (U) | Structural and systemic barriers to equitable maternity care  Women of refugee backgrounds experience complex, intersectional challenges after settlement that create multiple barriers to accessing equitable care. Some of these challenges are the result of broader structural and systemic barriers such as housing and migration policies. Strained, over-capacity and inadequately funded health systems also negatively impact the services available to provide equitable care to refugee background women. There is a particular need for more professional interpreting services and staff who can provide social support and care. Health professionals often lack time, resources, and capacity to deliver adequate care and health information, which contributes to the mismatches between service demand and delivery. This places an emotional and mental load on healthcare professionals which can lead to burnout. |  |
| Delays in access to care (U) |  |  |
| Challenges faced (U) |  |  |
| Vulnerable situation (C) |  |  |
| Pregnant refugees are a heterogeneous population facing multiple barriers to care (U) |  |  |
| System capacity for change (U) |  |  |
| Funding cuts created a confusing system which jeopardised care (C) |  |  |
| Socio-cultural and medical norms: differences between home and host country (U) |  |  |
| Syrian influx created additional strains on existing problems (U) |  |  |
| Relocations of asylum seekers (U) |  |  |
| Structural barriers to effective care (U) |  |  |
| Housing conditions (C) |  |  |
| Managerial discourse (C) |  |  |
| Experiences with maternity care system (priority needs) (U) |  |  |
| Language and interpreters (U) |  |  |
| Communication with clients (U) |  |  |
| Interpreting issues (U) |  |  |
| Using interpreters (NS) |  |  |
| Barriers to communication (U) |  |  |
| The MCH nurse role when working with families from a refugee background (C) |  |  |
| There is an imbalance between midwives’ responsibilities to care for the women and the resources available (shortage of resources) (U) |  |  |
| Organisational factors affecting Cross Cultural Worker Service provision (U) |  |  |
| The emotional cost of caring (U) |  |  |
| Understanding the needs, experiences and identities of refugee and migrant women (NS) |  |  |
| Challenges of caring for women who were unbooked (C) |  |  |
| The burden of traumatic stress on mothers and midwives (U) |  |  |
| Challenges in working with female patients from Ukraine (U) |  |  |
| Language barriers (U) |  |  |
| Cultural barriers (U) |  |  |
| Educational barriers (U) |  |  |
| Psychological barriers (U) |  |  |
| Challenges with caring for migrant women (U) |  |  |
| Doctor-centered system with minimal investment in the health care team (U) |  |  |
| Lack of service integration and continuity of care (U) |  |  |
| Low engagement during crisis – service providers’ burn out (U) |  |  |
| Pressures on healthcare professionals and community organisations in delivering care (U) |  |  |
| Structural inequities and the toll of the pandemic (U) |  |  |

| **Findings (n=114)** | **Categories and accompanying descriptions (n=5)** | **Synthesised finding 2 and explanatory statement** |
| --- | --- | --- |
| Life skills (U) | Personal agency and the factors that enable them  Shared decision-making is essential for women as women want to be part of the decision-making process concerning their health and the health of their baby. Personal agency is strengthened when women are provided with knowledge and information – ideally in peer-group settings – to learn about pregnancy, birth, and parenting. Gaining knowledge helps women feel more prepared, confident, and empowered to ask questions, seek information and navigate the healthcare system. The way health professionals interact with women can impact their autonomy in decision-making. When women experience negative interactions such as not being listened to, being excluded from the decision-making process, or having their choices overlooked, they may feel frustrated, disrespected, disempowered, and mistrust their health professional. Feeling heard, respected, and having their choices recognised by healthcare providers is vital to positive maternity care experiences. | Trauma-informed, culturally responsive, and continuity of care and carer are key to equity-oriented maternity care.  Approaches to maternity care systems that incorporate models of continuity of care and carer, trauma-informed care, and culturally responsive care strengthen communication and foster trusting, supportive relationships between women and their healthcare providers. Additionally, access to social support – both within and beyond the maternity care system – plays a vital role in supporting women as they navigate pregnancy, childbirth, and early parenting during settlement in a new country. |
| Promoting choice and control (U) |  |  |
| Learning together: informed, prepared, confident and reassured (U) |  |  |
| Gaining of knowledge (C) |  |  |
| Isolation and alienated knowledge (U) |  |  |
| Provider paternalism and women’s decision-making autonomy (U) |  |  |
| Personal agency (C) |  |  |
| Participatory decision-making (U) |  |  |
| Women as equal decision-makers in their perinatal care: The importance of consent and control (U) |  |  |
| Storytelling as an expression of self & shared power (U) |  |  |
| Language proficiency (U) |  |  |
| Positive drivers for use of prenatal care (U) |  |  |
| Provider paternalism and women’s decision-making autonomy (U) | Interpersonal relationships  When women feel acknowledged, heard, safe and that their healthcare needs are respected, trusting and supportive relationships between women and healthcare professionals can be formed. Continuity of care and carer also fosters the development of positive women-healthcare provider connections. Communication and language barriers can lead to feelings of powerlessness when women are unable to express their preferences or advocate for their needs. Experiences of covert racism such as health professionals showing frustration with women’s care decisions, displaying impatience or dismissing their concerns can undermine trust, and can even negatively impact women’s access to care. |  |
| The characteristics of health care providers (U) |  |  |
| Delayed hospital arrival during labour (U) |  |  |
| Dramatic experiences and disappointments with maternity care (U) |  |  |
| Communication and resource provision (U) |  |  |
| Feeling safe (U) |  |  |
| Trusting relationships: continuity of care and care provider (U) |  |  |
| Continuity of care and relationships with healthcare providers (U) |  |  |
| Not being asked or listened to (U) |  |  |
| Tragic experiences as a result of failures of the maternity care system (C) |  |  |
| Interaction with health staff (U) |  |  |
| Lack of communication, connection and culturally competent care (U) |  |  |
| Challenges in the hospital: communication and privacy (U) |  |  |
| Mistrust of Western health care (U) |  |  |
| Good experiences and expressed appreciation toward the maternity care system (U) |  |  |
| Being treated fairly and equally (C) |  |  |
| Women feeling accepted (U) |  |  |
| Value of relationality (U) |  |  |
| Support from health services (U) |  |  |
| Giving birth in a new and unfamiliar culture – the multicultural doula as a guide with the midwife (C) |  |  |
| The desired relationships with nurses (U) |  |  |
| Cultivating reciprocal curiosity (U) |  |  |
| Building foundations for belonging (U) |  |  |
| Interpersonal caring (U) |  |  |
| Support from health and social care provider systems and charitable organisations (U) |  |  |
| Experiences with maternal health-care providers (U) |  |  |
| Discrimination (U) |  |  |
| The importance of social support (C) | Loss and rebuilding of social support networks in a new country  Women experienced social isolation and loneliness during pregnancy, childbirth, and the postpartum period, particularly after resettling in a new country. The loss of traditional support systems – particularly family members and female kin – was deeply felt and longed for by the women, particularly during childbirth and the early postpartum period. Feelings of social isolation and loneliness were exacerbated during the postpartum period and were further heightened during the COVID-19 pandemic. Despite these challenges, women valued the practical and psychosocial support they received from refugee- specific organisations and community members that shared their language, cultural, and religious backgrounds. They also valued the support provided by non-medical staff working in maternity care systems such as doulas and bicultural workers. Women that participated in group pregnancy classes gained social and emotional support and connectedness from other mothers and were able to form friendships, share stories and experiences with them. These peer connections helped women to rebuild a sense of community and belonging and navigate motherhood in a new and unfamiliar environment. |  |
| Social and emotional support: sharing stories and experiences (U) |  |  |
| Social support (C) |  |  |
| Loss of informal support (U) |  |  |
| Community support and precarity (U) |  |  |
| Women and men’s experiences of being asked about social health issues (U) |  |  |
| Religious beliefs (U) |  |  |
| Support from health and social care provider systems and charitable organisations (U) |  |  |
| Enhancing connection to improve emotional wellbeing (U) |  |  |
| Family and female kin (U) |  |  |
| Social support and network (U) |  |  |
| Karen doulas: Patient advocates on the health care team (U) |  |  |
| Being alone and pregnant (U) |  |  |
| Isolation and alienated knowledge (U) |  |  |
| Being pregnant and dispersed (U) |  |  |
| Impacts of COVID-19 postnatal healthcare (U) |  |  |
| Anxiety and grief caused by COVID-19 (U) |  |  |
| Displacement and isolation worsen postpartum mental health (U) |  |  |
| Lack of communication, connection and culturally competent care (U) |  |  |
| Feeling alone and scared – safeguarded by the multicultural doula (U) |  |  |
| Needing to be looked after – cared for by the multicultural doula (U) |  |  |
| Not understanding the language – understanding with the multicultural doula (U) |  |  |
| Family support around the postpartum period (U) |  |  |
| Limitations of support due to separation from family (C) |  |  |
| Cultural safety during the pandemic (U) |  |  |
| Cultural differences (C) | Barriers at the interpersonal level  Health professionals encountered three main barriers when caring for refugee background women, this included cultural differences in care, communication challenges, and difficulties building trusting relationships. There were often differences in the way maternity care was provided between the women’s home country and country of settlement, which resulted in cross-cultural comparisons being made and differences in expectations of care. When tensions arose between traditional cultural practices and the care provided in Western healthcare settings, health professionals often faced moral and practical dilemmas in trying to adhere to organisational regulations and legislation while accommodating the women’s cultural needs. This can impact the development of trusting relationships. Many providers had a lack of understanding and limited knowledge of the cultural customs and beliefs around pregnancy, childbirth, and the postpartum period of the women they cared for. This sometimes led to feelings of frustration with the women, dismissing their concerns and expectations, and cultural stereotyping. Most health professionals did not speak the same language as the women and there was a lack of professional interpreter services available in hospitals and health services to bridge the communication and language gap. Having limited communication increases the likelihood of miscommunication, difficulty conveying emotions and preferences, reduces women’s autonomy and access to information and can negatively affect the provision and experience of care for both parties. Health professionals recognised continuity of care and carer as vital for the development of trusting relationships and women’s wellbeing but found it difficult to maintain as it was often hindered by factors outside of their control. |  |
| Cross cultural comparisons (U) |  |  |
| Understanding cultural difference (U) |  |  |
| Frustration with perceived Somali women’s resistance to obstetric interventions (U) |  |  |
| Agreeability and gratitude from “easy going patients” (U) |  |  |
| Language and interpreters (U) |  |  |
| Communication with clients (U) |  |  |
| Challenges in patient-provider communication (U) |  |  |
| Building trust (U) |  |  |
| Providers’ perception of mistrust by their Somali patients (C) |  |  |
| Continuity of care and relationships with healthcare providers (U) |  |  |
| Taking an individualised approach (NS) |  |  |
| Medical discourse (U) | Ways of working in maternity care  Health professionals tended to practice based on the medical model of care, focusing on the clinical needs of women only to the extent of the training they received. While many health professionals acknowledged the importance of providing continuity of care and culturally responsive care to women by the same provider, implementing these approaches proved easier in refugee-specific health services than in mainstream healthcare settings.  Health professionals frequently faced moral and practical dilemmas when trying to balance respect for women’s cultural beliefs and practices with organisational policies and clinical guidelines. The role of non-medical social support staff, such as doulas and bicultural workers, was recognised as pivotal to supporting and improving maternity care experiences for women and supporting healthcare professionals in their work. The shared culture, language and migration journeys fostered the building of trusting relationships. Women from refugee backgrounds have complex needs partly due to their traumatic experiences and require holistic, trauma-informed, and culturally competent care. Health professionals who were unfamiliar with refugee health and lacked specific training were unprepared and found it challenging caring for these women and meeting their complex and layered needs. This highlighted the need for and importance of increased education and training, support, and system-level changes in maternity care models. |  |
| Midwifery discourse (C) |  |  |
| Culturally and refugee responsive care (U) |  |  |
| Continuity of care (U) |  |  |
| Continuity of care and relationships with healthcare providers (U) |  |  |
| Taking an individualised approach (NS) |  |  |
| Service provision: models of care, access and appointments (C) |  |  |
| Interdisciplinary collaboration (U) |  |  |
| Working alongside peer supporters (U) |  |  |
| Interdisciplinary collaboration (C) |  |  |
| Strong partnerships (U) |  |  |
| Bicultural family mentors – the critical link (U) |  |  |
| Community support – role of doulas (U) |  |  |
| Cultural facilitator (U) |  |  |
| Improving the healthcare experience (U) |  |  |
| Access to other referral agencies (C) |  |  |
| Supporting access to health and community-based services (U) |  |  |
| Power dynamics at the intersection of community and clinical knowledge (C) |  |  |
| Finding our own ways of working together (U) |  |  |
| Karen doulas: Patient advocates on the health care team (U) |  |  |
| Supportive care (U) |  |  |
| Can I ask that? Another view of Karen perinatal preference and understanding (U) |  |  |
| Knowledge sharing (U) |  |  |
| Making connections (U) |  |  |
| Enhanced communication & rapport with providers (U) |  |  |
| Continuous individualised support (U) |  |  |
| The context of care (C) |  |  |
| Psychosocial care (NS) |  |  |
| Community support – role of doulas (U) |  |  |
| Improving access and quality of services (NS) |  |  |
| Non-judgemental support (U) |  |  |
| Social connectedness (U) |  |  |
| Creating safe spaces (U) |  |  |
| Prevalent mental health issues (U) |  |  |
| Vital elements of working with people who have experienced war and those with whom we do not share a language (U) |  |  |
| Experiences with immigrant clients (U) |  |  |
| Lack of service integration and continuity of care (U) |  |  |
| Cultural safety during the pandemic (U) |  |  |

| **Findings (n=32)** | **Categories and accompanying description (n=3)** | **Synthesised finding 3 and explanatory statement** |
| --- | --- | --- |
| Perceptions toward hospital and prenatal care (U) | The pre-migration experience  The maternity care needs of women are shaped by their pre-migration experience, particularly experiences of trauma, their experience of healthcare systems in their home country, and traditional cultural and religious beliefs and norms around pregnancy, labour and birth, and the postpartum period. For many women, seeking care from hospitals or health services was associated with illness. Pregnancy and childbirth were viewed as normal life events that did not warrant additional medical care or intervention. This often led to hesitancy in engaging with maternity care systems in countries of settlement. Women were also apprehensive about discussing their postpartum mental health concerns with healthcare professionals due to the cultural stigma surrounding mental health illnesses. Many women feared that disclosing this sensitive information could result in women being seen as unfit or incapable of caring for their children by the health professionals. | Women’s settlement experiences influence their transition to new healthcare systems  When caring for women from refugee backgrounds, it is important to take a holistic approach that considers their pre-migration experiences, cultural and religious beliefs, and the challenges they face during settlement. These factors shape their health-seeking behaviour, access to care, and how their maternity care needs and preferences are understood and approached. |
| Barriers to seeking support in formal settings (U) |  |  |
| It’s what women believe and do (U) |  |  |
| Experiences of maternity-related care (U) |  |  |
| Outright refusal of care (U) |  |  |
| Healthcare in country of origin (U) |  |  |
| Socio-cultural and medical norms: differences between home and host country (U) |  |  |
| Cultural norms and practices (U) |  |  |
| Can I ask that? Another view of Karen perinatal preference and understanding (U) |  |  |
| The role of intersecting identities in shaping maternal healthcare needs (U) |  |  |
| Stigma and fear discourage seeking postpartum mental health care (U) |  |  |
| Conceptions of pregnancy and childbirth, premigration experiences (C) |  |  |
| The impact of premigration experiences on women’s perceptions of health care needs (U) |  |  |
| Religious beliefs (U) |  |  |
| Resistance to health practices (U) |  |  |
| Intentionally not seeking or misleading prenatal care (U) |  |  |
| Views on postpartum blues/depression (U) |  |  |
| The fear of caesarean section (U) |  |  |
| The importance of cultural and religious practices (U) |  |  |
| Need for and fear of a caesarean delivery (U) |  |  |
| Maternal healthcare experiences (U) |  |  |
| Multidimensionality effects knowledge, preferences, and expectations (U) |  |  |
| Women’s understanding of health and wellbeing during pregnancy and after childbirth (U) |  |  |
| Challenges as a refugee (U) | Challenges experienced during the settlement period  Women experienced a variety of challenges after arriving in a new country. These challenges included learning a new language, adjusting to foreign climates, navigating new systems such as healthcare, and coping with social isolation and the absence of familiar support systems. For many women, these challenges were compounded by the demands of pregnancy, caring for a newborn, or looking after other children. Balancing the stress of settlement, and roles as mothers and wives created an additional layer of stress of women, making it more difficult for them to adjust to life in a new country. |  |
| The role of intersecting identities in shaping maternal healthcare needs (C) |  |  |
| Adaptation and its influence on pregnancy, birth and postpartum experiences (U) |  |  |
| Understanding the US health care system (C) |  |  |
| Displacement and isolation worsen postpartum mental health (U) |  |  |
| Complexity of the US health system combined with unfamiliarity contributes to lack of confidence (C) |  |  |
| New opportunities (U) | New opportunities and looking ahead  In their countries of settlement, many women experienced a shift in traditional gender roles during pregnancy, childbirth, and the postpartum period. Whilst female relatives usually accompanied and provided support to birthing and postpartum mothers in their home countries, husbands were often present during labour and childbirth in the countries of settlement and took on a more active role in the postpartum period caring for the women. This practice may have been considered unusual in the women’s home countries; however, it was welcomed by women and some of their husbands. Women appreciated the increased involvement of their husbands throughout pregnancy, childbirth, and postpartum. Women also expressed a desire to build a future in their new country and wanted to support other women of refugee backgrounds in the future navigate the maternity care system. |  |
| Changing roles of men (U) |  |  |
| Building a future (U) |  |  |

*The total number of findings includes findings that have been mapped to more than one category

## Appendix E: ConQual Summary of Findings

| Synthesised Finding | Type of research | Dependability | Credibility | ConQual Score | Comments |
| --- | --- | --- | --- | --- | --- |
| Structural and systemic factors shape equity-oriented maternity care provision and access  Healthcare professionals and women of refugee background face a range of structural and systemic enablers and barriers that influence the provision and access of culturally responsive and equitable maternity care. These factors are interrelated and span individual, interpersonal, organisational and policy levels, shaping care experiences at outcomes in complex ways. The main structural and systemic barrier shared between healthcare professionals and women is rooted in language and communication. | Qualitative | High (No change) | Moderate (Downgrade one level) | Moderate | No change to dependability score as majority of studies (28/50) scored 4 or 5 out of 5 for dependability criteria. Credibility score was downgraded by 1 as there was a mix of unequivocal and credible findings |
| Trauma-informed, culturally responsive, and continuity of care are key to equity-oriented maternity care  Approaches to maternity care systems that incorporate continuity of care, trauma-informed care, and culturally responsive care strengthen communication and foster trusting, supportive relationships between women and their healthcare providers. Additionally, access to social support – both within and beyond the maternity care system – plays a vital role in supporting women as they navigate pregnancy, childbirth, and early parenting during resettlement in a new country. | Qualitative | High (No change) | Moderate (Downgrade one level) | Moderate | No change to dependability score as majority of studies (25/46) scored 4 or 5 out of 5 for dependability criteria. Credibility score was downgraded by 1 as there was a mix of unequivocal and credible findings |
| Women’s resettlement experiences influence their transition to new healthcare systems  When caring for women of refugee background, it is important to take a holistic approach that considers their pre-migration experiences, cultural and religious beliefs, and the challenges they face during resettlement. These factors shape their health-seeking behaviour, access to care, and how their maternity care needs and preferences are understood and approached | Qualitative | High (No change) | Moderate (Downgrade one level) | Moderate | No change to dependability score as majority of studies (15/19) scored 4 or 5 out of 5 for dependability criteria. Credibility score was downgraded by 1 as there was a mix of unequivocal and credible findings |
